# CTCF is a DNA-tension-dependent barrier to cohesin-mediated loop extrusion

**DOI:** 10.1038/s41586-023-05961-5

**Published:** 2023-04-19

**Authors:** Iain F. Davidson, Roman Barth, Maciej Zaczek, Jaco van der Torre, Wen Tang, Kota Nagasaka, Richard Janissen, Jacob Kerssemakers, Gordana Wutz, Cees Dekker, Jan-Michael Peters

**Affiliations:** 1 Research Institute of Molecular Pathology, Vienna BioCenter, Vienna, Austria; 2 Department of Bionanoscience, Kavli Institute of Nanoscience Delft, Delft University of Technology, Delft, Netherlands; 3 Present Address: Children’s Cancer Research Institute, St Anna Kinderkrebsforschung, Vienna, Austria

**Keywords:** Single-molecule biophysics, DNA

## Abstract

In eukaryotes, genomic DNA is extruded into loops by cohesin^1^. By restraining this process, the DNA-binding protein CCCTC-binding factor (CTCF) generates topologically associating domains (TADs)^2,3^ that have important roles in gene regulation and recombination during development and disease^1,4–7^. How CTCF establishes TAD boundaries and to what extent these are permeable to cohesin is unclear^8^. Here, to address these questions, we visualize interactions of single CTCF and cohesin molecules on DNA in vitro. We show that CTCF is sufficient to block diffusing cohesin, possibly reflecting how cohesive cohesin accumulates at TAD boundaries, and is also sufficient to block loop-extruding cohesin, reflecting how CTCF establishes TAD boundaries. CTCF functions asymmetrically, as predicted; however, CTCF is dependent on DNA tension. Moreover, CTCF regulates cohesin’s loop-extrusion activity by changing its direction and by inducing loop shrinkage. Our data indicate that CTCF is not, as previously assumed, simply a barrier to cohesin-mediated loop extrusion but is an active regulator of this process, whereby the permeability of TAD boundaries can be modulated by DNA tension. These results reveal mechanistic principles of how CTCF controls loop extrusion and genome architecture.

## Abstract (web summary)

CTCF is sufficient to block loop extruding cohesin in a DNA tension dependent manner, and induces loop extrusion direction switching and loop shrinkage.

## Main

The folding of genomic DNA by cohesin has important roles in chromatin organization, gene regulation and recombination^1^. Cohesin belongs to the structural maintenance of chromosomes (SMC) family of ATPase complexes that can extrude DNA into loops, an activity that has been reconstituted in vitro for cohesin, condensin, and SMC5/SMC6 (refs. ^9–14^). Cohesin also performs a second function by mediating sister-chromatid cohesion.

In individual cells, loops are located at variable positions, suggesting that loops are dynamic structures of which most are in the process of being extruded^15–17^. However, in cell-population measurements, patterns emerge that reveal that most loops are formed within TADs^16,18,19^. CTCF is located at TAD boundaries^18,19^ and is required for their formation and for cohesin accumulation at these sites^2,3,20^. CTCF has unstructured N- and C-terminal regions that flank 11 zinc fingers, several of which recognize an asymmetric DNA sequence and therefore position CTCF directionally on the DNA^21,22^. Most CTCF-binding sites are oriented in convergent orientations so that CTCF’s N termini face the interior of TADs, suggesting that CTCF functions as an asymmetric boundary to cohesin-mediated loop extrusion^23–25^. Consistent with this possibility, the N terminus of CTCF can bind to cohesin^26^ and is required for TAD insulation and loop anchoring at these sites^26–29^.

Several mechanisms have been suggested for how CTCF might prevent loop extrusion across TAD boundaries (reviewed previously^8^), namely, as a physical barrier (roadblock); by binding to cohesin; by preventing the release of cohesin from DNA, by promoting the replacement of cohesin’s ATPase-activating subunit NIPBL by its inactive counterpart PDS5; by directly inhibiting cohesin’s ATPase activity; and by promoting entrapment of DNA inside a ring structure that is formed by three of cohesin’s subunits^30^. It has also been proposed that CTCF converts cohesin into an asymmetrically extruding enzyme by stalling loop extrusion at the CTCF-bound site while allowing cohesin to continue reeling DNA into the loop only from the TAD interior^26,31,32^. However, it remains unresolved which of these proposed mechanisms is used by CTCF and whether CTCF is sufficient for blocking loop extrusion by cohesin. Answering these questions is of great importance, as CTCF is required for controlling enhancer–promoter interactions^1^, nuclear reprogramming^6^, recombination of antigen receptor genes^4,5^ and the timing of DNA replication^33^, and because CTCF mutations have been implicated in tumorigenesis^7^. CTCF boundaries are also sites at which replicated DNA molecules are connected by cohesin complexes, which mediate cohesion^34^.

## CTCF characterization in vitro

**Fig. 1:**
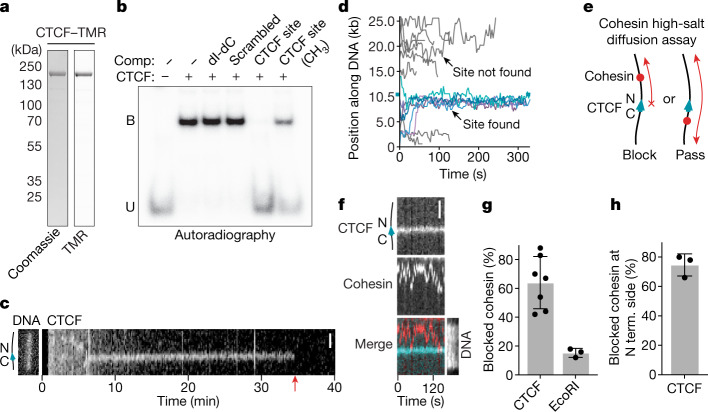
CTCF is a directional barrier to cohesin diffusion on DNA. **a**, Coomassie staining of recombinant CTCF after analysis using SDS–PAGE. Tetramethylrhodamine (TMR) was visualized by epi-green excitation. Gel source data are provided in Supplementary Fig. 1. **b**, Autoradiograph of EMSA. CTCF was incubated with a ^32^P-labelled DNA containing a CTCF-binding site. Where indicated, the reactions were supplemented with excess unlabelled competitors (comp.). dI-dC, poly(2′-deoxyinosinic-2′-deoxycytidylic acid); B, bound; U, unbound. Gel source data are provided in Supplementary Fig. 1. **c**, Example of TMR-labelled CTCF diffusing on DNA. Non-specifically bound CTCF molecules diffuse randomly and dissociate rapidly. At 5.5 min, a CTCF molecule binds to DNA and diffuses until encountering the CTCF-binding site at 6 min. Scale bar, 2 µm. The red arrow indicates the timepoint at which CTCF bleached or dissociated. **d**, Superposition of individual TMR-labelled CTCF-diffusion events. Events in which CTCF localized to its binding site at position 10452 bp (cyan tick) are shown in blue (*n* = 6). DNA-binding events in which CTCF did not localize to its binding site are shown in grey. *n* = 11. **e**, Illustration of the cohesin diffusion assay. **f**, Example of cohesin diffusion that is blocked by CTCF. Cohesin and CTCF were labelled with Alexa660 (red) and TMR (blue), respectively. Sytox Green DNA stain was introduced into the flow cell at the end of the experiment. Scale bar, 2 μm. **g**, The fraction of blocking events in which cohesin encountered CTCF or EcoRI(E111Q). Data are mean ± s.d. from 7 (*n* = 264) and 3 (*n* = 106) independent experiments, respectively. **h**, The fraction of blocked events in which cohesin diffused along the DNA between the tether point and the N-terminal (N term.) side of CTCF. Data are mean ± s.d. from 3 (*n* = 48) independent experiments. In the remaining 25% of events, cohesin diffused between the tether and the C-terminal side of CTCF. Sample sizes refer to biological replicates. Source Data

**Extended Data Fig. 1:**
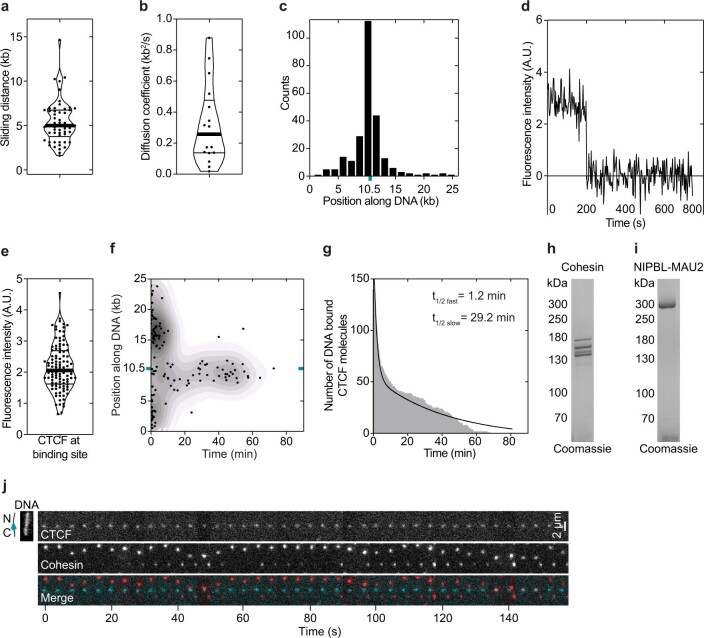
Recombinant CTCF characterization. **a**, Distance (kb) travelled by TMR labelled CTCF molecules while diffusing before encountering the CTCF binding site or dissociating. The thick line denotes the median; thin lines denote quartiles. N = 54. **b**, Diffusion coefficient of diffusing TMR labelled CTCF molecules. The thick line denotes the median; thin lines denote quartiles. N = 17. **c**, Position of DNA bound TMR labelled CTCF following a brief wash step. The CTCF binding site (cyan tick) is at position 10,452 bp out of 26,123 bp. N = 251. The orientation of the DNA was determined using end-labelling by TetR as shown in Extended Data Fig. 2f. **d**, Time trace of Alexa 660 (A660)-labelled CTCF signal bound at its DNA binding site bleaching in one step. **e**, Fluorescence intensity of A660 labelled CTCF signals at the CTCF binding site. N = 104. The thick line denotes the median; thin lines denote quartiles. **f**, Residence time of TMR labelled CTCF on DNA. The CTCF binding site (cyan tick) is at position 10,452 bp out of 26,123 bp. N = 140. **g**, Residence time of TMR-labelled CTCF on DNA from (f) plotted as a histogram. Bi-exponential decay curve was fitted using Prism. **h-i**, Coomassie staining of recombinant cohesin and NIPBL-MAU2 after SDS-PAGE. For gel source data, see Supplementary Fig. 1. **j**, Example of cohesin diffusion blocked by CTCF. Cohesin and CTCF were labelled with A660 and TMR, respectively. Sytox Green DNA stain was introduced into the flow cell at the end of the experiment. This data is identical to main Fig. 1f except it is formatted as a montage rather than as a kymograph. Source Data

To obtain insights into how CTCF controls cohesin, we developed in vitro assays in which CTCF–cohesin interactions can be visualized on DNA at the single-molecule level in real time. We first analysed how CTCF finds its DNA consensus sequence. Consistent with previous reports^35,36^, recombinant human CTCF (Fig. 1a) bound specifically to DNA oligonucleotides containing a single CTCF-binding site in electrophoretic mobility shift assays (EMSAs) in a manner that was reduced by DNA methylation (Fig. 1b). We introduced this CTCF-binding site into linear 26.1 kb DNA molecules, tethered these at both ends to glass surfaces in flow cells, stained with Sytox Green and imaged the DNA molecules using highly inclined and laminated optical sheet (HILO) microscopy. After introduction of fluorophore-labelled CTCF, both immobile and mobile CTCF foci were observed at various positions along the DNA (Fig. 1c,d and Extended Data Fig. 1a). CTCF foci at the CTCF-binding site were detectable for much longer than those elsewhere, where CTCF proteins often dissociated rapidly unless they arrived at the CTCF-binding site while diffusing along DNA (diffusion coefficient = 0.32 ± 0.1 kb^2^ s^−1^; Fig. 1c,d and Extended Data Fig. 1b). These results indicate that CTCF finds its DNA-binding site by facilitated diffusion. Most CTCF foci that were not located at the CTCF-binding site were removed by a brief salt wash, in contrast to those at the CTCF-binding site (Extended Data Fig. 1c). Fluorescence intensity and photobleaching analysis indicated that these remaining CTCF molecules were monomers (Extended Data Fig. 1d,e). Once bound to their binding sites, the mean residence time of CTCF molecules was around 29 min (Extended Data Fig. 1f,g), which is longer than most^37–42^ but not all^40^ in vivo estimates, and longer than an in vitro measurement described in a recent preprint^43^. It is possible that additional factors, such as the action of other chromatin-bound proteins, might promote CTCF unbinding in cells.

## CTCF is a polar barrier to cohesin

**Extended Data Fig. 2:**
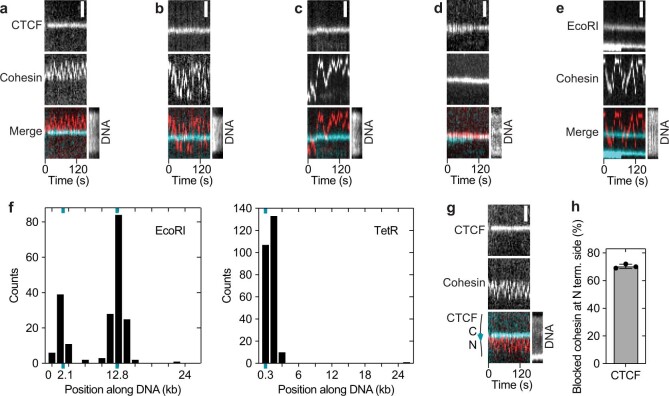
Cohesin diffusion assay characterization. **a–d**, Examples of cohesin diffusion on DNAs with CTCF bound at its binding site. Cohesin was labelled with Alexa 660 (red). CTCF was labelled with tetramethylrhodamine (TMR) (cyan). Sytox Green DNA stain was introduced into the flow cell at the end of the experiment. Scale bar, 2 μm. (a) Example of cohesin diffusion blocked by CTCF. (b) Example of cohesin diffusing past CTCF multiple times within the imaging timeframe. (c) Example of cohesin diffusing past CTCF in one direction only. Example of cohesin diffusing past CTCF in one direction only. This behaviour was observed very infrequently (2 ± 3% of N = 264 events). This could be because cohesin-CTCF encounters were recorded after the system has reached equilibrium and so all the single-pass events had occurred before we could image them. It is unknown why some cohesin molecules were able to pass CTCF multiple times (Extended Data Fig. 2b). (d) Example of cohesin-CTCF colocalization. **e**, Example of cohesin diffusing past TMR-labelled EcoRI^E111Q^. **f**, Positions of DNA bound (left) Janelia Fluor 646-labelled EcoRI^E111Q^ and (right) TMR-labelled TetR, which were flowed into flow cells at the end of diffusion experiments to determine the DNA orientation and hence the orientation of the CTCF binding site at position 10,452 bp. EcoRI restriction sites were present at positions 2,177 bp and 12,802 bp out of 26,123 bp. N = 201. Six TetO sequences were present at positions 40–274 bp. N = 251. **g**, As in main Fig. 1f, except using a DNA in which the CTCF site was inverted. **h**, Fraction of blocked events that diffused on the DNA between the tether point and the N terminal side of CTCF using the DNA template as used in (g) (mean ± SD (N = 48) from 3 independent experiments). Source Data

Next, we analysed how CTCF interacts with cohesin that diffuses along DNA. For this purpose, we used an assay in which cohesin associates with DNA in a high-salt-resistant manner that is sensitive to cohesin and DNA cleavage^44^, suggesting that, under these conditions, cohesin entraps DNA topologically and moves along DNA as has been proposed for cohesive cohesin^34,45^. We observed that CTCF frequently blocked diffusion of recombinant human cohesin (64 ± 18%; mean ± s.d.), while the remaining cohesin traversed CTCF multiple times (Fig. 1e–g and Extended Data Figs. 1h–j and 2a–d). By contrast, EcoRI(E111Q) rarely blocked cohesin (15 ± 3%; Fig. 1g and Extended Data Fig. 2e). To determine the orientation of the CTCF molecules that had blocked cohesin translocation, we post-labelled the DNA molecules with a marker protein that binds to one of their ends (Extended Data Fig. 2f). This revealed that 75 ± 8% (mean ± s.d.) of the blocked cohesin complexes faced the N-terminal side of CTCF (Fig. 1h). This can be attributed to the orientation of CTCF, as inversion of its binding site reversed this blocking behaviour (Extended Data Fig. 2g,h). As diffusing cohesin binds to DNA in a manner that is consistent with entrapment^44^, which is believed to be the interaction mode by which cohesin mediates cohesion^45^, this suggests that CTCF contributes to the accumulation of cohesive cohesin at TAD boundaries^34^.

## CTCF is a polar barrier to DNA looping

**Extended Data Fig. 3:**
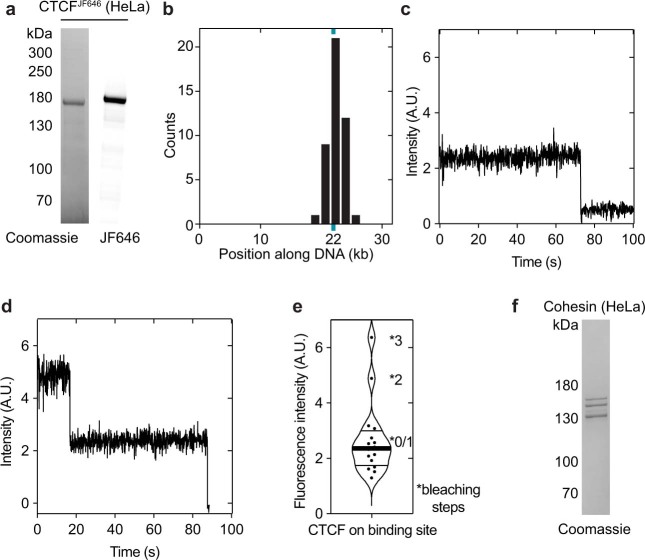
HeLa CTCF characterization. **a**, Coomassie staining of HeLa CTCF after SDS-PAGE. JF646 was visualized by epi-red excitation. For gel source data, see Supplementary Fig. 1. **b**, Position of DNA bound JF646-labelled CTCF following a wash step with a buffer supplemented with 220 nM Sytox Orange. The CTCF binding site (cyan tick) is at position 9,667 bp out of 31,767 bp. N = 251. **c**, Time trace of JF646-labelled CTCF signal bound at its DNA binding site bleaching in one step. **d**, Time trace of JF646-labelled CTCF signal bound at its DNA binding site bleaching in two steps. **e**, Fluorescence intensity of JF646-labelled CTCF signals at the CTCF binding site. The thick line denotes the median; thin lines denote quartiles. N = 16. **f**, Coomassie staining of HeLa cohesin after SDS-PAGE. For gel source data, see Supplementary Fig. 1. Source Data

To test whether CTCF also acts as a barrier to loop-extruding cohesin, we introduced a single CTCF site at position 9.7 kb in a 31.8 kb DNA, such that CTCF’s N terminus would face the longer end of the DNA. We tethered both ends of these molecules to the surfaces of flow cells and stained them with Sytox Orange. We then bound CTCF purified from HeLa cells (Extended Data Fig. 3a–e) to these DNA molecules and introduced HeLa cohesin (Extended Data Fig. 3f), recombinant NIPBL–MAU2 (Extended Data Fig. 1i) and ATP.

**Fig. 2:**
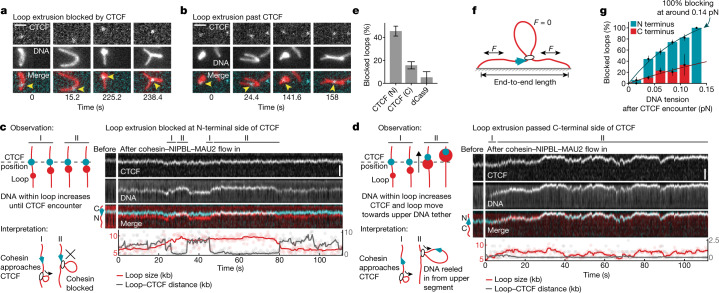
CTCF is a direction- and tension-dependent barrier to cohesin-mediated DNA loop extrusion. **a**,**b**, Examples of loop extrusion blocked by (**a**) or passing (**b**) CTCF (cyan) labelled with Janelia Fluor 646 (JF646). DNA loops (red) were visualized by Sytox Orange and perpendicular buffer flow. Scale bar, 2 µm. **c**, Cohesin-mediated DNA loop extrusion encountering N-terminally oriented JF646-labelled CTCF (cyan). Growth of DNA loop stops after encountering CTCF at around 30 s and around 50 s. Scale bar, 2 µm. **d**, The same as **c**, but for a passing event. CTCF passes into the loop at 70 s and translocates with it. Scale bar, 2 µm. **e**, The fraction of loop-extrusion events blocked after encountering N- or C-terminally oriented CTCF or dCas9. Data are mean ± 95% binomial confidence interval. *n* = 119, 115 and 19 from 13, 3 and 3 independent experiments for N-terminal, C-terminal and dCas9 encounters, respectively. The force range between 0.04 and 0.08 pN was best covered and was therefore chosen to compare the overall blocking efficiency (Extended Data Fig. 5c,d). **f**, The DNA tension at the moment of the encounter was calculated by the amount of DNA outside the loop and the DNA end-to-end length (Supplementary Note). **g**, The loop-extrusion blocking probability of N- or C-terminally oriented CTCF depends on DNA tension. Data are mean ± 95% binomial confidence interval. The solid lines are fits of the form 1 − exp(−*F*/*F*_0_), which were used to compute the force at which 100% blocking is achieved (N-terminal encounters: *P*_block_(*F*) = 147(1 − e^−^^*F*/0.125 pN^); C-terminal encounters: *P*_block_(*F*) = 115(1 − e^−*F*/0.357 pN^). *n* per bin for N-terminal (N) and C-terminal (C) encounters: 0–0.015 pN: 17 (N) and 12 (C); 0.015–0.026 pN: 75 (N) and 77 (C); 0.026–0.05 pN: 72 (N) and 53 (C); 0.05–0.072 pN: 89 (N) and 34 (C); 0.096–0.119 pN: 40 (N) and 6 (C); and 0.119–0.142 pN: 3 (N) and 0 (C). The bin for C-terminal encounters at the highest DNA tension regime is not shown owing to insufficient observations (*n* < 3). Sample sizes refer to biological replicates from 13 independent experiments for N-terminal encounters and 3 independent experiments for C-terminal encounters. Source Data

**Extended Data Fig. 4:**
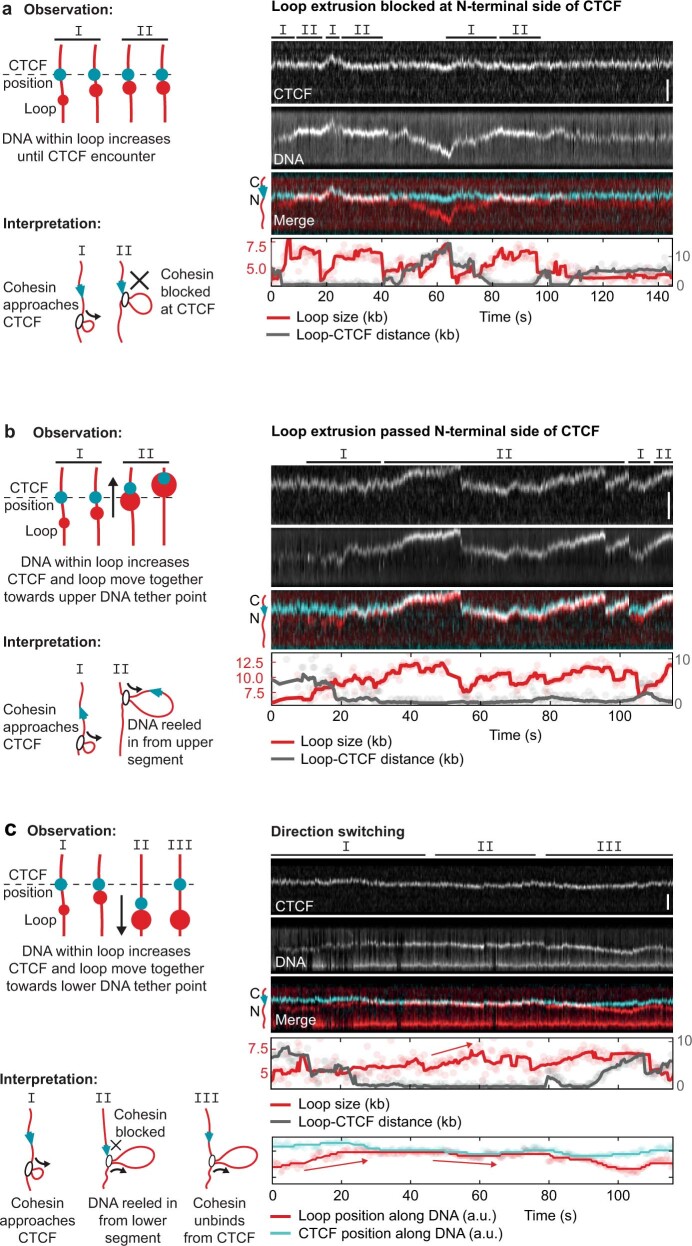
Additional examples of loop extrusion blocking, passing and direction switching upon encountering CTCF. **a–c**, (Left panels) observation and interpretation illustrations of (right panels) kymographs of cohesin-mediated DNA loop extrusion encountering N-terminally oriented CTCF (cyan) labelled with Janelia Fluor 646 (JF646). DNA loops were visualized by Sytox Orange stain. Scale bar, 2 µm. (a) Growth of the DNA loop stops upon encountering CTCF at timepoints ~ 12 – 18 s, 22 – 40 s and 82 – 95 s. (b) The DNA loop continues to grow upon encountering CTCF at 31 s, and CTCF passes into the loop and translocates with it. (c) The growing loop encounters CTCF at 28 s. CTCF and the growing DNA loop move towards the lower DNA tether point, indicating extrusion on the side facing away from CTCF.

After buffer flow perpendicular to the DNA axis, CTCF could be detected either near the base of (Fig. 2a) or within (Fig. 2b) DNA loops, suggesting that it functioned as a barrier to loop extrusion in some but not all cases. To analyse this behaviour quantitatively, we monitored loop extrusion in the absence of buffer flow, whereby loop formation results in the appearance of a bright spot on the DNA that increases in intensity over time. Tracking and quantification of loop position and size as well as of CTCF position permitted the classification of encounters between cohesin-mediated DNA loops and CTCF (Fig. 2c,d; additional examples are shown in Extended Data Fig. 4a,b and videos and animated illustrations are shown in Supplementary Videos 1 and 2). These experiments revealed that N-terminally oriented CTCF blocked the progression of loop extrusion in 45 ± 9% (mean ± s.d.) of encounters (Fig. 2c,e and Extended Data Fig. 4a,b), whereas the blocking efficiency was reduced to 16 ± 7% (mean ± s.d.) when we used DNA molecules in which the orientation of the CTCF-binding site had been inverted and on which cohesin therefore encountered CTCF’s C terminus (Fig. 2d,e). By contrast, the control protein dCas9, which has a larger mass (180,000 Da) than CTCF–Halo–Flag (118,600 Da) blocked loop extrusion in only 5 ± 10% (mean ± s.d.) of encounters (Fig. 2e), consistent with the finding that cohesin can readily traverse non-interacting DNA-bound particles during loop extrusion^46^.

**Extended Data Fig. 5:**
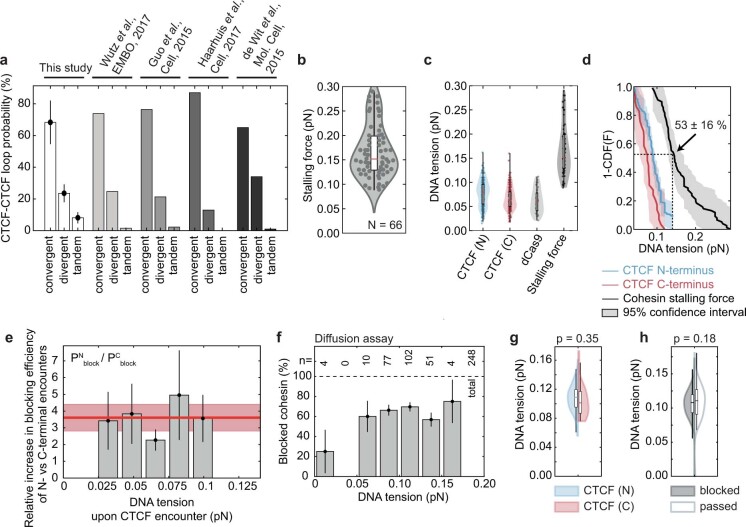
Stalling force of cohesin and force sampling for encounters with the N-/C-terminus of CTCF and dCas9. **a**, Combinatorial loop extrusion blocking efficiency at a pair of CTCF sites oriented in a convergent (><), tandem (>> and <<), and divergent (<>) manner. The percentages were obtained by multiplying the blocking probability of N- and C-terminal encounters in the force range 0.04-0.08 pN, as depicted in Fig. 2e, and normalizing to 100% (see Supplementary Note). Bar heights denote mean values. Error bars denote the error propagation after multiplication, given the 95% binomial confidence interval as depicted in Fig. 2e. The relative fraction of CTCF-anchored loops that we obtained from the single-molecule experiments are compared to published values extracted from Hi-C data^3,63–65^. **b**, Stalling force of cohesin. horizontal line median; boxes extend to the quartiles and the whiskers show the range of the data (median-1.5* interquartile range (IQR); median+1.5*IQR). Data from 2 independent experiments. **c**, The DNA tension measured at encounters of loop-extruding cohesin with the N- and C-terminus of CTCF and dCas9. The stalling force values from panel (b) is shown for comparison. N = 297, 184, 37, 66 for CTCF (N), CTCF (C), dCas9 and the stalling force measurements, respectively. **d**, The empirical survival function (1-CDF) of the data shown in panel c. Thick line represents the mean; shaded areas represent 95% confidence intervals. At the DNA tension of complete stalling at the CTCF N-terminus, 0.14 pN, the survival function decays to 53 $\pm$ 16%, i.e. if loops would be halted by reaching the stalling force alone, one would expect ~53% of loops to exceed the DNA tension of 0.14 pN, which was not observed (compare blue line for stalling at the CTCF N-terminus and Fig. 2g). **e**, Ratio of the N-terminal and C-terminal blocking probabilities. N-terminal encounters block loop extrusion 3.6 ± 0.8 -fold (The bar height denotes the mean, error bars denote the error propagation after multiplication, given the 95% binomial confidence interval as depicted in Fig. 2g) more often than encounters from CTCF’s C-terminal side, independently of DNA tension. N per bin for N-terminal (n) and C-terminal (c) encounters: 0.025-0.0415 pN: 70 (n), 72 (c); 0.0415-0.058 pN: 81 (n), 67 (c); 0.058-0.075 pN: 84 (n), 30 (c); 0.075-0.091 pN: 20 (n), 14 (c); 0.091-0.1075 pN: 40 (n), 6 (c); 0.119-0.142 pN: 3 (n), 0 (c). Sample sizes refer to biological replicates. **f**, Fraction of blocked molecules in the cohesin diffusion assay as a function of DNA tension (note that the DNA tension is constant in diffusion assays since no DNA loop is being extruded). The bar height denotes the mean, error bars denote the error propagation after multiplication, given the 95% binomial confidence interval. **g**, DNA tension of DNA molecules on which diffusing cohesin was blocked by N-terminally oriented CTCF (left; N = 74 from 2 independent experiments) or by C-terminally oriented CTCF (right; N = 27 from 5 independent experiments). Statistical significance was assessed by a 2-sided 2-sample Kolmogorov-Smirnoff test. **h**, Violin plot of DNA tension for DNA molecules on which diffusing cohesin was blocked by CTCF (left; N = 161 from 7 independent experiments) or repeatedly passed CTCF (right; N = 88 from 7 independent experiments). Statistical significance was assessed by a 2-sample Kolmogorov-Smirnoff test. Thick horizontal lines on boxplots denote median values, the box extends from the lower to upper quartile values and whisker limits denote the range of data within 1.5 times the interquartile range from the median. Source Data

These results indicate that monomeric CTCF, despite its relatively small mass and Stokes radius (5 nm for the N terminus of CTCF)^47^, is sufficient to block loop extrusion by cohesin in a directional manner, possibly because the N terminus of CTCF can bind to cohesin^26^. Notably, the N- and C-terminal blocking frequencies of 45% and 16% observed in our experiments can explain very well in vivo estimates of how frequently loops are detected between CTCF sites oriented in a convergent, divergent or tandem manner (Extended Data Fig. 5a), suggesting that CTCF may be solely responsible for determining how frequently loops are anchored at these differently oriented sites. While performing these experiments, we also observed that loops occasionally translocated along the DNA without increasing in size (Fig. 2c,d), a behaviour that is reminiscent of forms of condensin that are defective in the DNA-binding site formed at the interface between the HAWK subunit YCG1 and the kleisin BRN1 (refs. ^10,31^).

## CTCF is a DNA-tension-dependent barrier

Notably, we observed that the CTCF-blocking efficiency for loop-extruding cohesin depends on the tension in the DNA that is reeled in. As DNA molecules are tethered at both ends in our assay, loop extrusion continuously shortens the non-extruded parts of the DNA molecules and therefore increases their tension until this tension exceeds the stalling force of loop extrusion^10^ (Fig. 2f). We noticed that larger loops and loops extruded from DNAs with a longer end-to-end length tended to be stalled more efficiently by CTCF compared with those formed from less stretched DNA. As both scenarios coincide with larger tension in the unextruded part of the DNA, we tethered DNA molecules to the surface of flow cells with various degrees of ‘slack’, performed CTCF loop-extrusion blocking assays and calculated the tension that DNA molecules experienced when cohesin encountered CTCF.

**Extended Data Fig. 6:**
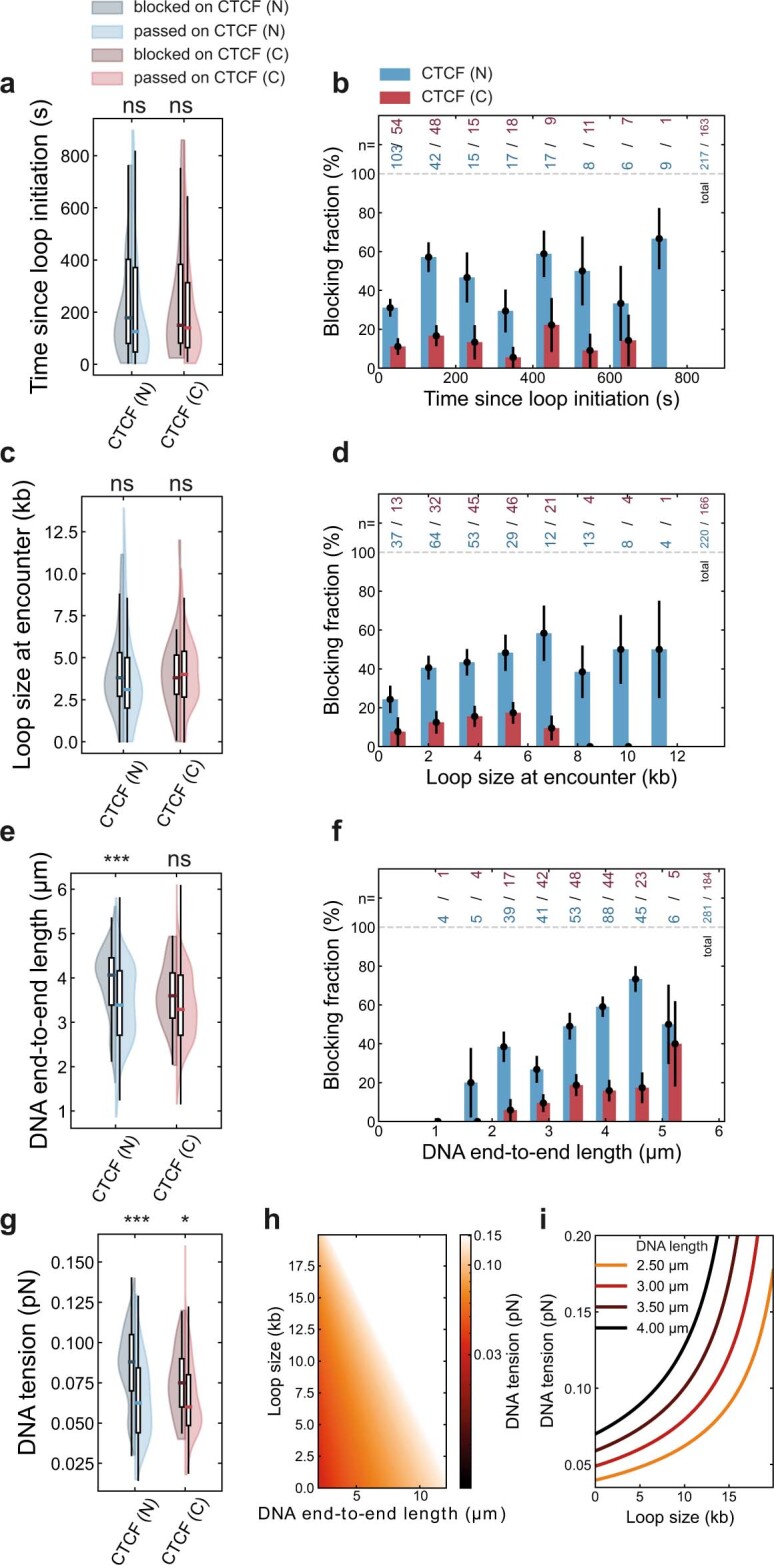
Effect of time since loop extrusion initiation, loop size, DNA end to end length and DNA tension on the loop extrusion blocking probability of CTCF. **a**, Time since loop extrusion initiation and N-terminal (blue) and C-terminal (red) CTCF encounters for events which blocked (left part of violin, dark shading) and did not block loop extrusion (right part of violin, light shading). The horizontal line is the median; boxes extend to the quartiles and the whiskers show the range of the data (median-1.5* interquartile range (IQR); median+1.5*IQR). NTD: p = 0.14; CTD: p = 0.89. **b**, CTCF blocking fraction for N-terminal (blue) and C-terminal (red) encounters for binned times between loop initiation and CTCF encounter. The number of data points per bin is shown on top. Error bars on bar plots denote 95% confidence intervals. **c,d**, as for (a,b) but for the loop size at encounter (NTD: p = 0.08; CTD: p = 0.90). Error bars on bar plots denote 95% confidence intervals. **e,f**, as for (a, b) but for the DNA end-to-end length (NTD: p = 5.05 x 10^−5^; CTD: p = 0.09). Error bars on bar plots denote 95% confidence intervals. DNAs with a higher end-to-end length are under higher DNA tension due to entropic effects. **g**, as for (a) but for the DNA tension (NTD: p = 1.38 x 10^−9^; CTD: p = 0.02). For a binned representation of the CTCF blocking probability against DNA tension, see Fig. 2g. Thick horizontal lines on boxplots denote median values, the box extends from the lower to upper quartile values and whisker limits denote the range of data within 1.5 times the interquartile range from the median. Error bars on bar plots denote 95% confidence intervals. **h**, Calculated DNA tension for values of DNA end-to-end length and loop size. The colour scale shows white for DNA tension values of ≥ 0.15 pN (see Supplementary Information). **i**, Cross-sections through the two-dimensional representation in (h) for specific values of DNA end-to-end length. Even without an extruded loop (loop size = 0 kb), the tethering of the DNA to the surface at the given end-to-end lengths contributes to the DNA tension. For example, a 31.8 kb DNA construct tethered with an end-to-end length of 4 μm (black line) results in a DNA tension of ~0.07 pN. Statistical significance was assessed by a two-sided Mann-Whitney U test without multiple comparison adjustments.

The efficiency of CTCF’s barrier activity indeed very strongly correlated with increased DNA tension (Fig. 2g and Extended Data Fig. 6g). Notably, our data indicate that CTCF does not block loop extrusion by cohesin at all when no force is applied, whereas CTCF blocks loop extrusion increasingly when tension is applied to the DNA, with CTCF reaching a blocking efficiency of 100% at approximately 0.14 pN. This tension is close to 0.15 pN, the median value of the force required to stall loop extrusion itself (Extended Data Fig. 5b–d). Encounters from the C-terminal side showed a similar trend, that is, blocked loop extrusion more frequently at higher tension, but with much lower blocking frequencies. By contrast, the ratio of blocking efficiencies of N-terminal versus C-terminal encounters (3.6 ± 0.8-fold (mean ± s.d.)) was unaffected by DNA tension (Extended Data Fig. 5e). Blocking at high DNA tensions was not due to the stalling force alone, as our data indicate that only N-terminal but not C-terminal encounters cause complete blocking at 0.14 pN (Fig. 2g). Furthermore, in the absence of CTCF, 53 ± 16% of DNA loops continued to grow at DNA tensions above 0.14 pN (Extended Data Fig. 5d), whereas encounters with N-terminally oriented CTCF displayed complete blocking at this DNA tension (Fig. 2g).

We also tested other parameters that might induce the blocking of loop extrusion by the N-terminally oriented CTCF. The time elapsed between the initiation of loop-extrusion initiation and encounter with CTCF, and the loop sizes at the time of encounter were not significantly different between blocking and passing events (Extended Data Fig. 6a–d). However, blocking events were more frequently observed on DNA with a larger end-to-end length (Extended Data Fig. 6e,f), which can be attributed to these DNA molecules experiencing a larger DNA tension even in the absence of an extruded loop (that is, an ‘offset tension’ of, for example, approximately 0.07 pN at 4 μm end-to-end length; Extended Data Fig. 6h,i), and the blocking force at the encounter with CTCF is therefore more readily reached after loop extrusion.

**Extended Data Fig. 7:**
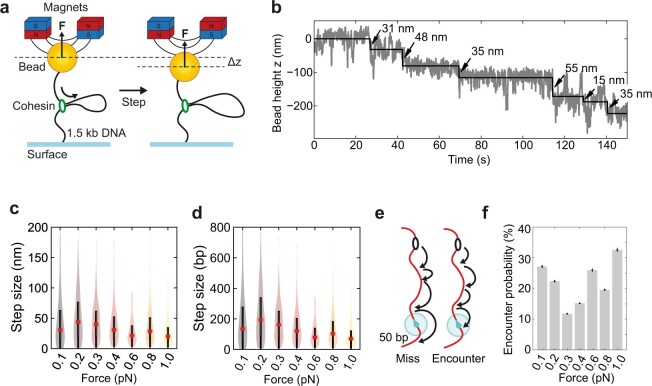
The force-dependent step size of cohesin loop extrusion does not solely explain the observed force dependence of CTCF blocking loop extrusion. **a**, Magnetic tweezers setup to observe individual loop extrusion steps by human cohesin, depending on the applied force, based on^48^. The change in bead height Δz corresponds to steps by cohesin. **b**, Example magnet tweezer trace showing stepwise changes in bead height in the presence of cohesin, NIPBL-MAU2 and ATP. Line denotes steps fitted using the step-finding algorithm. **c**, Step sizes in nanometres as measured by Magnetic Tweezer experiments, for various applied forces ranging from 0.1 pN to 1 pN. The horizontal line is the median; boxes extend to the quartiles and the whiskers show the range of the data (median-1.5* interquartile range (IQR); median+1.5*IQR). N = 100, 128, 168, 116, 148, 338, 270 from left to right from 2 independent experiments. **d**, Step sizes versus force from (c), but converted to base pairs. The median, quartiles and data range are shown as described in (c). **e**, Simulation setup: starting from a randomly chosen binding position along DNA, cohesin takes steps along DNA, which are sampled from the measured step size distribution. An ‘encounter’ is considered if cohesin comes within 50 bp of CTCF. Under the lenient assumption that the CTCF N-terminus is unstructured and may be approximated by a freely jointed chain, its radius of gyration R_G_ is estimated using the N_K_ = 268 amino acids from the N-terminus to zinc finger 126, with a contour length of l_K_ ~0.4 nm per amino acid^66^, resulting in R_G_ = N_k_l_K_2/6 ~7 nm^67^. This distance corresponds to roughly 20 bp, given the contour length of a basepair of 0.3 nm. A threshold of 50 bp was thus conservatively chosen because the CTCF N-terminus may be as long as 14 nm but is likely more compact due to folding of the CTCF N-terminus. The simulations thus likely represent an upper limit of the encounter probability. **f**, Simulated encounter probability of cohesin and CTCF (mean ± 95% binomial confidence interval; N = 500 independent simulations). Note that the encounter probability does not exceed ~40%, even at the smallest step size distribution (measured at 1 pN). In contrast, the blocking probability of N-terminal encounters of cohesin and CTCF increases from 0 to 100% within 0-0.14 pN (Fig. 2g). Force-dependent step sizes of cohesin can thus not solely explain the observed N-terminal blocking probability. We therefore suspect that DNA tension increases the blocking efficacy of CTCF by other mechanisms, such as by reducing not only cohesin’s step size but also the frequency with which it takes steps, thus providing more time for CTCF and cohesin to bind to each other; or by reducing thermal fluctuations of DNA^49^, which could reduce the space that CTCF has to explore to find cohesin. It is also conceivable that cohesin’s ‘motor’ activity can overcome the low 1 µM binding affinity of CTCF-cohesin interactions^26^ more easily at low DNA tension than at high tensions, which are close to the stalling force of loop extrusion, and at which cohesin has to generate higher forces to extrude DNA. Finally, DNA tension could also change cohesin’s responsiveness to CTCF by influencing how cohesin performs loop extrusion^68^. Source Data

To test whether the blocking of cohesin-driven loop extrusion by N-terminally oriented CTCF could relate to the ability of loop-extruding cohesin to ‘step over’ CTCF, we measured cohesin’s step size during loop extrusion using magnetic tweezers. These measurements showed that cohesin on average takes large steps of about 40 nm (100–200 bp) on DNA and that the step size decreases when DNA tension increases (Extended Data Fig. 7a–d), as also observed for condensin^48^. We tested in simulations whether cohesin might encounter CTCF more frequently at higher DNA tension because cohesin is less likely to step over CTCF as the extrusion steps become smaller. However, this hypothesis was not supported by our simulations (Extended Data Fig. 7e,f). We therefore suspect that DNA tension increases the blocking efficiency of CTCF by other mechanisms, such as reducing the step frequency at increased tension, which allows more time for CTCF–cohesin binding; decreasing the thermal fluctuations of DNA^49^, which may reduce the space that CTCF has to explore to find cohesin; or that cohesin’s weak motor activity can more easily overcome the low binding affinity of CTCF–cohesin interactions^26^ at low DNA tension compared with at high tension (Extended Data Fig. 7f). Irrespective of these interpretations, our results indicate that local changes in DNA tension that could be caused by nucleosome assembly, transcription, DNA replication, supercoiling or other processes can affect genome architecture by modulating the permeability of TAD boundaries. As loop extrusion is sensitive to DNA tension^10^ but diffusion is not, we hypothesized that the DNA tension dependence of CTCF’s barrier activity might only occur after encountering loop-extruding cohesin but not for diffusing cohesin. Indeed, we found that CTCF’s ability to block diffusing cohesin is independent of DNA tension (Extended Data Fig. 5f–h). Thus, although CTCF acts as a barrier to diffusing cohesin, it can block loop-extruding cohesin only at higher DNA tensions.

## Transient loop anchoring by CTCF

**Fig. 3:**
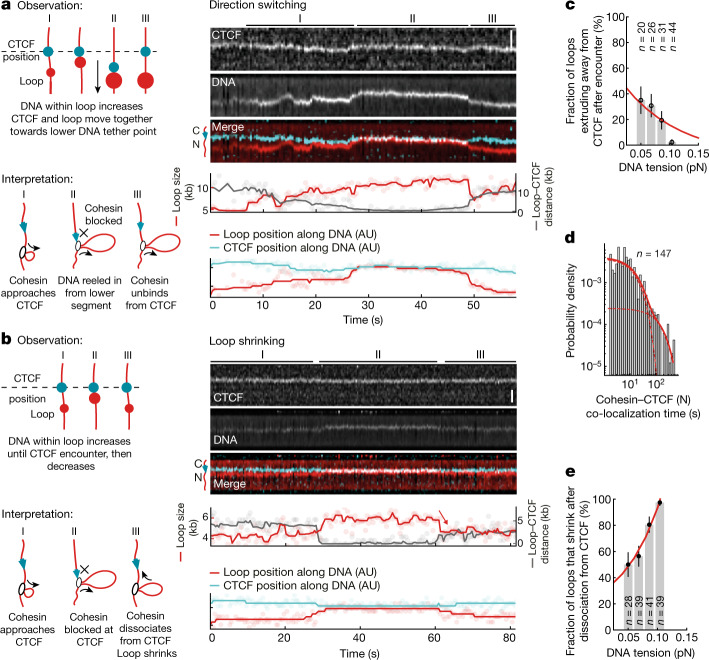
CTCF changes the direction of cohesin-mediated loop extrusion or induces loop shrinkage, depending on the DNA tension. **a**,**b**, Observation and interpretation illustrations (left) of kymographs of cohesin-mediated DNA loop extrusion encountering N-terminally oriented, JF646-labelled CTCF (cyan) (right). DNA loops (red) were visualized by Sytox Orange. Scale bars, 2 µm. In **a**, the growing loop encounters CTCF at 28 s. CTCF and the growing DNA loop move towards the lower DNA tether point, indicating extrusion on the side facing away from CTCF. In **b**, the growth of the DNA loop stops after encountering CTCF at around 29 s. The DNA loop shrinks after dissociation from CTCF at approximately 60 s. AU, arbitrary units. **c**, The fraction of loops extruding away from CTCF versus the DNA tension at the moment of encounter. Data are mean ± 95% binomial confidence interval; 13 independent experiments. **d**, The co-localization time of encounters between cohesin and the N terminus of CTCF. The fit denotes a two-component exponential distribution with rate constants *k*_1_ = 0.06 s^−1^ and *k*_2_ = 0.006 s^−1^ (*τ*_1_ ≈ 17 s and *τ*_2_ ≈ 167 s; data are from 13 independent experiments). The dashed lines represent the individual components of the two-component exponential distribution. The solid line represents the final two-component exponential distribution. **e**, The fraction of loops that shrink after release from CTCF versus DNA tension at the moment of encounter. Data are mean ± 95% binomial confidence interval; 13 independent experiments. Sample sizes refer to biological replicates. Source Data

**Extended Data Fig. 8:**
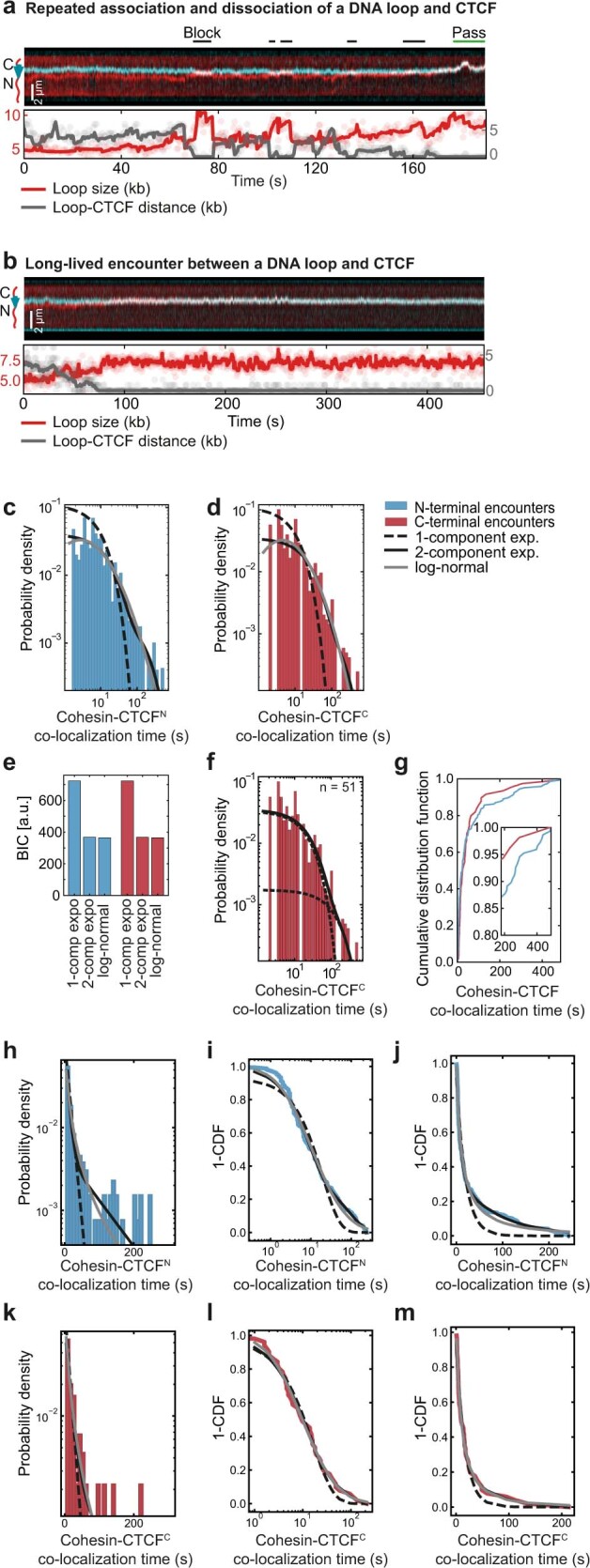
Cohesin-CTCF residence time characterization. **a**, Example of repeated approaching of CTCF (cyan) by cohesin, blocking of further loop extrusion and dissociation of the cohesin-CTCF interaction. Cohesin passes CTCF at the end of the kymograph. DNA loops (red) were visualized by Sytox Orange stain. Scale bar, 2 µm. **b**, Example of a growing loop encountering CTCF (cyan), stalling and co-localizing until the end of image acquisition. DNA loops (red) were visualized by Sytox Orange stain. Scale bar, 2 µm. **c**, Co-localization times of cohesin for the encounters from the N- and **d**, C-terminal side of CTCF (N = 147 and N = 51 for N- and C-terminal encounters, respectively). The distributions are fitted to a mono-exponential, bi-exponential and log-normal distribution. **e**, Bayesian Information Criterion (BIC) for the three models on N- and C-terminal encounters. Notably, both a bi-exponential as well as a log-normal distribution fit the distributions equally well. The parameters of the log-normal fits of the form (xσ√2π)^−1^ exp((ln(x)-μ)^2^/(2σ^2^)) are μ = 3 s, σ = 1.5 s for N-terminal and μ = 3 s, σ = 1.3 s for C-terminal encounters. **f**, The residence time of encounters between cohesin and CTCF’s C-terminus is well described by a bi-exponential distribution with rate constants k_1_ = 0.04 s^−1^ and k_2_ = 0.01 s^−1^ (τ_1_ ~ 25 s and τ_2_ ~ 100 s). **g**, Cumulative distribution function of the cohesin-CTCF co-localization time for N- (blue) and C-terminal (red) encounters. Inset: magnified view of co-localization times ≥ 3 min. **h**, data from panel (c) on a linear x-axis. **i**, The data in panels (c) and (h) plotted as 1-CDF (Cumulative Distirbution Function) on a logarithmic x-axis. **j**, the data in panel (i) plotted on a linear x-axis. **k–m**, as panels (h–j) for encounters of cohesin with C-terminally oriented CTCF. Source Data

To analyse the fate of loops that were blocked by CTCF, we first determined how long CTCF and loops co-localize under conditions in which the loop size was constant (that is, where loop extrusion stalled after an encounter). We frequently observed brief (tens of seconds) and repeated encounters between loops and CTCF (Extended Data Fig. 8a and Supplementary Video 3) as well as occasional encounters that lasted for several minutes (Extended Data Fig. 8b). The distribution of CTCF–loop interaction times after stalling events was well described by a biexponential distribution, indicating the existence of two populations with mean CTCF–loop association times of 16 s and 167 s (Fig. 3d and Extended Data Fig. 8c–m).

In contrast to CTCF’s blocking function, the CTCF–loop association time was largely unaffected by CTCF orientation (Extended Data Fig. 8c–m). It is conceivable that the infrequent C-terminal blocking events that we observed represent occasions on which cohesin in fact encountered CTCF’s N terminus after passing over its C terminus. The results indicate that CTCF interacts with cohesin mostly transiently (more than 85% of encounters lasted less than 3 min; Extended Data Fig. 8g), which is similar to the lifetime that has been measured for particular loops in cells^17^. However, longer-lived loops have been predicted to exist for up to several hours^32,50^. As we did not observe such prolonged co-localization of CTCF and loops, additional proteins may be required to anchor loops for such long time periods, for example, the PDS5 proteins, which are also required for TAD boundaries in cells^3^.

## CTCF can switch the direction of looping

It has been speculated that cohesin switches from symmetric to one-sided asymmetric extrusion at TAD boundaries at which loop ‘stripes’ or ‘flames’ have been detected in Hi-C experiments^23,26,31,32^. We therefore analysed whether a change in extrusion symmetry could be observed when cohesin encounters CTCF. Although cohesin appears to extrude symmetrically in vitro^9,11,12^, we observed that cohesin frequently reels in DNA first from one side and then the other, switching direction multiple times (a detailed analysis of this bidirectional extrusion will be reported in a separate study (Barth, R. et al., manuscript in preparation)). We therefore analysed whether CTCF can trigger a switch of the direction of loop extrusion. To investigate this, we monitored the size of DNA loops and their position relative to CTCF after encounters that had blocked loop extrusion.

**Extended Data Fig. 9:**
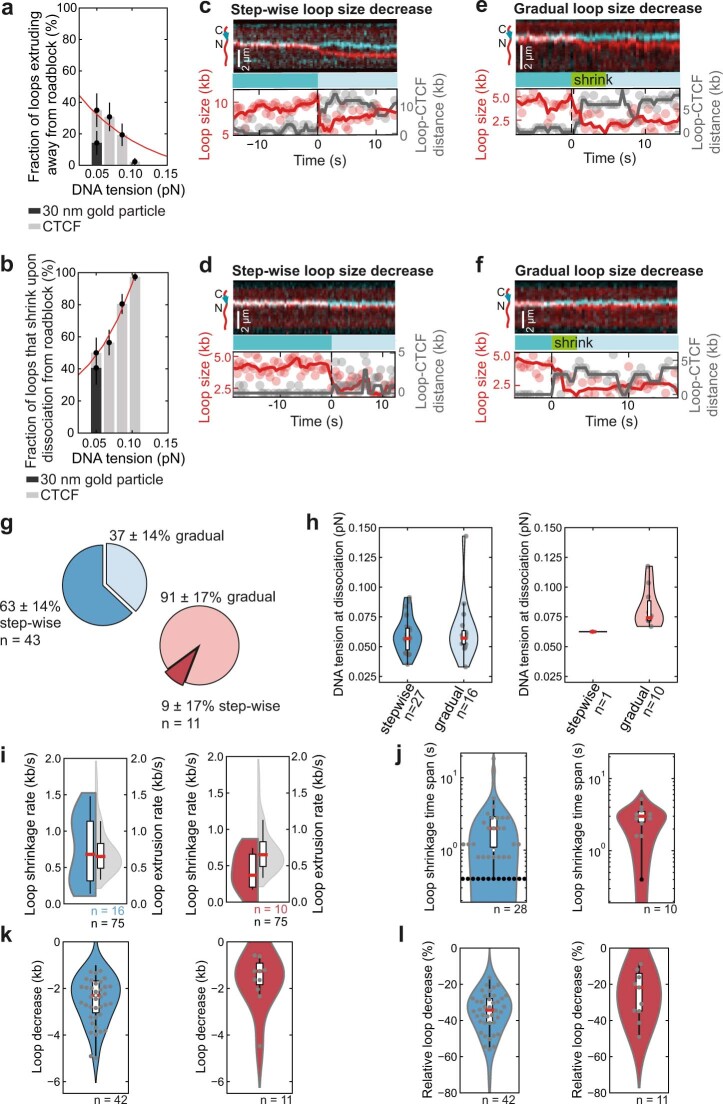
Characterization of direction switching and loop shrinkage following encounters between cohesin and CTCF or gold nanoparticles. **a**, The fraction of loops extruding on the side facing away from CTCF (grey bars) or 30 nm gold nanoparticles (black bar; 14 ± 8% [mean ± 95% binomial confidence interval]). CTCF data is replotted from Fig. 3c. Encounters with gold nanoparticles over a force range of 0.02-0.05 pN were reanalysed from^46^ (N = 21 biological replicates from 2 independent experiments). **b**, The fraction of loops which shrink upon release from CTCF (grey bars) or 30 nm gold nanoparticles (black bar; 41 ± 10% [mean ± 95% binomial confidence interval]) versus DNA tension at the moment of encounter. CTCF data is replotted from Fig. 3e. Encounters with gold nanoparticles over a force range of 0.02-0.05 pN were reanalysed from^46^ (N = 22 biological replicates from 2 independent experiments). **c-d**, Examples of step-wise and **e–f**, continuous loop shrinkage upon dissociation of cohesin from CTCF. Scale bar, 2 µm. Related to Fig. 3b. **g**, The fraction of step-wise and continuous loop shrinkage for encounters from the N-terminal (blue) and C-terminal (red) side (mean ± 95% binomial confidence interval). **h**, DNA tension for loops which shrink step-wise or gradually. There is no statistically significant difference in DNA tension between the two modes (p > 0.05, 2-sided 2-sample Kolmogorov–Smirnov test). **i**, Loop shrinkage rate, in comparison to cohesin loop extrusion rate (grey), and **j**, distribution of shrinkage time spans. Black dots represent step-wise shrinkage events that happen within one imaging time interval, i.e. 0.4 s. **k**, Absolute and **l**, relative loop size decrease for N- and C-terminal encounters in blue and red, respectively. Thick horizontal lines on boxplots denote median values, the box extends from the lower to upper quartile values and whisker limits denote the range of data within 1.5 times the interquartile range from the median. Data for N-/C-terminal encounters were collected from 13 and 3 independent measurements, respectively. Source Data

At low DNA tension, we observed events in which CTCF indeed switched the direction of cohesin’s loop-extrusion activity. Cohesin approached CTCF by reeling in the intervening DNA and then, after an encounter with CTCF, it began to reel in DNA from the other direction while remaining bound to CTCF (Fig. 3a,c, Extended Data Fig. 4c and Supplementary Video 4). A control experiment with gold nanoparticles that were tethered to DNA as artificial roadblocks^46^ reversed the direction of loop extrusion 2.6× less frequently at low DNA tension (Extended Data Fig. 9a), suggesting that this ability may be a specific property of CTCF. This effect can potentially explain the appearance of ‘stripes’ and ‘flames’ at TAD boundaries.

Notably, at higher DNA tension, CTCF did not switch the direction of loop extrusion (Fig. 3c) but, instead, loops tended to shrink in size after release from CTCF (Fig. 3b,e, Extended Data Fig. 9c–l and Supplementary Video 5). In most cases, loops decreased in size within a single step (that is, within the imaging frame speed of 0.4 s; Extended Data Fig. 9c,d,g,h) but, in some cases, loops shrunk gradually over several seconds at a rate similar to that of loop extrusion (Extended Data Fig. 9e–j). In both cases, loops did not disrupt completely but were reduced in size by several kb and on average lost 35% of looped DNA (Extended Data Fig. 9k,l). Such loop shrinkage could be observed with similar frequencies when cohesin collided with artificial roadblocks on DNA (Extended Data Fig. 9b), suggesting that this may be a general response of cohesin to encountering barriers on DNA, irrespective of specific binding of the roadblock to cohesin. Its physiological relevance and whether it represents a reversal of the loop-extrusion mechanism or ‘slippage’ of DNA from the loop remains to be investigated, but it is interesting that the gradual shrinkage occurred at a similar rate as loop extrusion.

## Discussion and conclusions

Our results indicate that CTCF molecules find their cognate binding sites by facilitated diffusion and, once bound to them, are sufficient as monomers to block passively diffusing cohesin complexes, possibly reflecting how DNA-entrapping cohesive cohesin accumulates at TAD boundaries^34^. CTCF is also a barrier to actively loop-extruding cohesin, presumably reflecting how CTCF establishes TAD boundaries. As predicted from Hi-C experiments, CTCF performs this function asymmetrically with its N terminus blocking cohesin almost fourfold more efficiently compared with at its C terminus. Notably, this function is regulated by the tension of the DNA that CTCF and cohesin are bound to, implying that genomic processes that alter DNA tension will modulate the permeability of CTCF boundaries and, therefore, the length of chromatin loops extruded by cohesin.

**Extended Data Fig. 10:**
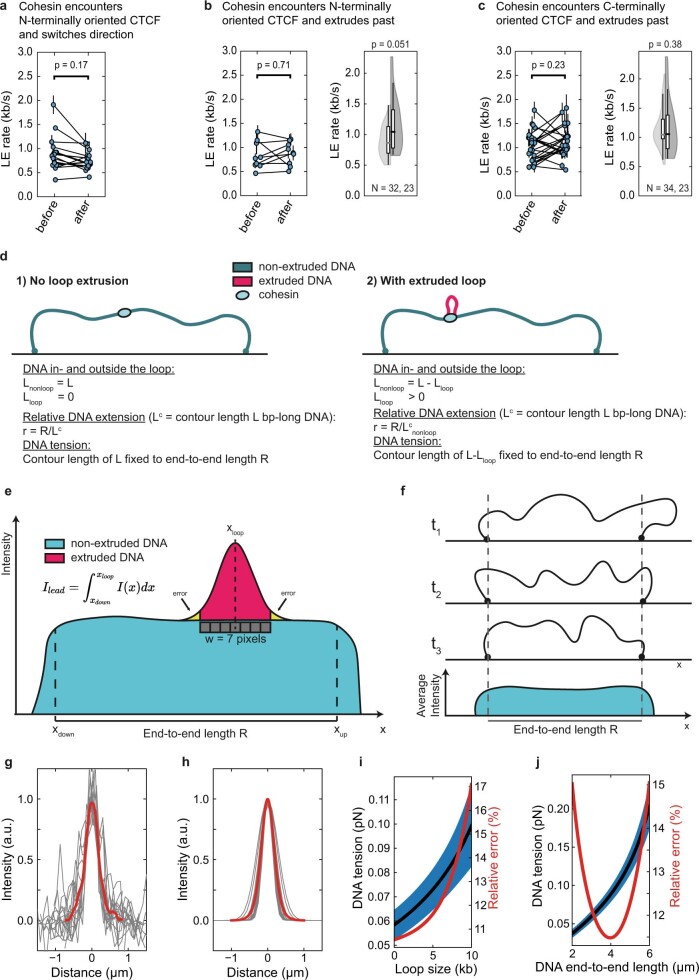
The loop extrusion rate does not change after encounter of cohesin with CTCF. **a**, Loop extrusion (LE) rate before and after encounter with N-terminally oriented CTCF when cohesin was blocked at CTCF and then switched extrusion direction to extrude away from it (see e.g. Fig. 3a and Supplementary Video 4). Statistical significance was assessed by a 2-sided Wilcoxon rank-sum test (mean $\pm$ SEM; N = 22). **b**, As for (a) but for events where cohesin passed over N-terminally oriented CTCF (mean $\pm$ SEM; N = 9). For events where the time between onset of LE and encounter with CTCF was too short to measure the LE rate, the LE rate was determined after passage and compared to the LE rate in the absence of CTCF (split violin plot on the right). For the latter, statistical significance was assessed by a 2-sided 2-sample Kolmogorov-Smirnoff test. **c**, as for (b) for events where cohesin passed over C-terminally oriented CTCF (mean $\pm$ SEM; N = 38). Error bars on individual data points denote the standard deviations of determined loop extrusion rates in moving 11-frame windows (4.4 s) during the duration of loop extrusion before encounter. Thick horizontal lines on boxplots denote median values, the box extends from the lower to upper quartile values and whisker limits denote the range of data within 1.5 times the interquartile range from the median. Sample sizes (N) refer to biological replicates from 13 independent experiments for N-terminal and 3 independent experiments for C-terminal encounters. **d–j**, Illustrations of loop size and DNA tension determination, and DNA tension error estimation (see Supplementary Note). (d) In the absence of loop extrusion, a DNA molecule of length L bp is tethered to a surface with end-to-end distance R. The relative DNA extension is thus computed as the ratio of the end-to-end distance R and the contour length of the entire DNA molecule. In the presence of an extruded DNA loop, the non-extruded part of the DNA is effectively shortened by the loop size L_loop_, while the DNA inside the loop does not experience any tension in the absence of buffer flow. The relative extension is now computed as ratio of the (constant) end-to-end length R and the contour length of the non-extruded DNA, which has the size L_nonloop_ = L − L_loop_. An increasing loop progressively shortens the non-extruded part of the DNA, giving rise to an increasing tension on the non-extruded DNA due to a fixed end-to-end length R. (e) Illustration of the DNA intensity profile along its long axis. The DNA intensity appears slightly larger than its real end-to-end length due to convolution of the DNA intensity with the microscope point spread function, which is corrected for by the peak peeling algorithm to determine the DNA end-to-end length. The lead intensity is determined as the integrated intensity between one of the DNA ends and the loop position (which corresponds to the peak position of the looped DNA intensity. A 7-pixel window around the loop position is summed and corrected by the intensity contributing in this window from the non-extruded DNA. However, the loop intensity is underestimated due to truncation of the integration outside the range [x_loop_ − w/2, x_loop_ + w/2] (yellow area). (f) At low end-to-end length, the flexibility of DNA might yield a DNA intensity beyond the DNA’s tether points. (g) Cross-sections around point-emitters (grey; N = 15) centred at their maximum value and mean trace (red). (h) Gaussian fits the single traces (grey) and mean fit (red) centred at their maximum value. The average Gaussian width was found to be σ = 180 ± 13 nm. (i) DNA tension with absolute (black line and blue area correspond to mean and error of the DNA tension) and relative (red) error of the DNA tension over loop sizes from L_loop_ = 0 to 10 kb at fixed end-to-end length R = 3.5 μm. Error bars denote the estimated error, also represented as a red line on the right y-axis. (j) Analogous to (i) for a fixed loop size of L_loop_ = 5 kb and varying end-to-end length R. Error bars denote the estimated error, also represented as a red line on the right y-axis. Source Data

A preprint published after submission of this Article reported that CTCF bound to an array of four CTCF-binding sites is an impermeable barrier to DNA compaction mediated by cohesin^43^. The reasons for this higher blocking activity compared with our study are unclear, but it is possible that the number of CTCF molecules that cohesin encounters affects its ability to bypass. Furthermore, the authors used a continuous buffer flow, which induces a high degree of DNA tension (which can be estimated to be around 0.5 pN), presumably hindering the passing of CTCF. The authors also reported that cohesin slowed its DNA compaction rate when encountering N-terminally oriented CTCF and accelerated when encountering C-terminally oriented CTCF. We however did not observe significant changes to the rate of loop extrusion when loop-extruding cohesin passed over N-terminally or C-terminally oriented CTCF (Extended Data Fig. 10b,c) or when it encountered the N-terminally oriented CTCF and then switched direction to continue extruding away from the CTCF (Extended Data Fig. 10a).

**Fig. 4:**
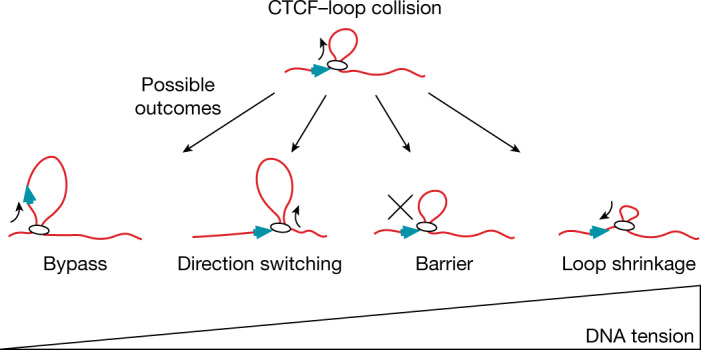
DNA tension affects the outcome of CTCF–loop collisions. At low DNA tension, CTCF is frequently incorporated into the growing DNA loop. At higher DNA tensions, CTCF promotes loop-extrusion direction switching, blocks loop extrusion and, at the highest DNA tensions, induces loop shrinkage.

Our data indicate that encounters with CTCF can alter cohesin’s loop-extrusion activity in at least three different ways (Fig. 4): it can block loop extrusion; it can switch its direction, that is, cause cohesin to reel in DNA from the opposite side as before; and it can lead to a process in which loop formation is reverted as the loop starts shrinking rather than growing. The observation that TADs detected by Hi-C are ‘filled’ with chromatin loops that are not anchored at both TAD boundaries may therefore reflect not only the presence of nascent loops that have not been fully extruded yet, as has been assumed so far, but also the existence of ‘shrunk’ loops that had already reached TAD boundaries but were switched there into a ‘reverse’ mode by CTCF. It is conceivable that such a backtracking process is used as a failsafe mechanism for enabling repeated interactions between specific genomic regions in cases in which these remained unproductive after their first encounter during forward loop extrusion, for example, during V(D)J recombination of antigen receptor genes^4,5^.

Although indirect effects of CTCF on cohesin—for example, inhibition of WAPL or promotion of PDS5 binding at the expense of NIPBL—may enhance the establishment of a barrier to loop extrusion as detected in cell-population measurements (reviewed previously^8^), our experiments indicate that these effects are not strictly required. Together, our findings reveal that CTCF controls cohesin and therefore genome architecture through multiple modes. Our results will provide the basis for future mechanistic and physiological studies of CTCF’s key functions in gene regulation, recombination and tumorigenesis.

## Methods

### DNA constructs for use as substrates in the cohesin diffusion assay

DNA fragments containing a single HighOc1 CTCF-binding site^51^ (TCAGAGTGGCGGCCAGCAGGGGGCGCCCTTGCCAGA) were generated by PCR using Phusion Hot Start DNA polymerase (NEB, M0535S) and inserted into the plasmid pPlat (25,754 bp) at the FspAI (Thermo Fisher Scientific, ER1661) restriction site in either forward or reverse complement orientation using Gibson assembly^52^. The constructs were then linearized using the restriction enzyme SpeI (New England Biolabs, R3133S) and biotinylated as previously described^44^.

### DNA constructs for use as substrates in the loop-extrusion assay

We prepared two constructs of 31.8 kb length containing a CTCF site placed asymmetrically ~9.7 kb from one end, which enables discrimination of the orientation of the DNA construct on the basis of the binding position of CTCF. One construct was oriented such that the N terminus of CTCF points towards the longer end of the DNA (plasmid 121; used for N-terminal encounters) and the motif direction of the other construct was reversed (plasmid 128; used for C-terminal encounters). Plasmid 121 was generated using plasmids 64, 66, 67, 69, 118 and 71 (see Supplementary Table 1 for a complete list of the intermediate vectors and primers used). Plasmid 128 was generated using plasmids 64, 66, 124, 69, 118 and 71 (Supplementary Table 1). Plasmids 121 and 128 were constructed using Golden Gate cloning, using BsaI-HFv2 as the type-2 restriction enzyme (NEB, E1602). Intermediate vectors (64, 66, 67, 124, 69, 118 and 71) were generated using Gibson assembly and traditional (restriction enzyme based) cloning techniques (Supplementary Table 1) (NEB, E2621 Gibson mix; NEB, M0515 Q5 polymerase).

Biotin-containing handles were generated by a PCR reaction with primers JT337 (biotin-GACCGAGATAGGGTTGAGTG, IDT) and JT338 (biotin-CAGGGTCGGAACAGGAGAGC, IDT) on plasmid 18 pBluescript SK+ (Stratagene), using GoTaq 2 (Promega, M7845). This results in a 1,238 bp PCR fragment, which was cleaned up using Promega Wizard SV Gel and PCR Cleanup System (Promega, A9282). Fresh plasmids 121 and 128 were purified using the Qiafilter plasmid midi kit (Qiagen, 12243). After purification, the plasmids were cut with both XhoI and NotI-HF and biotin handles were cut with either XhoI or NotI-HF. The digested products were mixed together with around a 10× molar excess of the biotin handle over the linearized plasmid. Ligation was performed using T4 DNA ligase (NEB, M0202L) overnight at 16 °C and heat-inactivated the next morning for 20 min at 65 °C. The resulting 31.8 kb DNA construct was cleaned up using the ÄKTA pure system, with a homemade gel-filtration column containing approximately 46 ml of Sephacryl S-1000 SF gel filtration medium (Cytiva) in TE + 150 mM NaCl_2_. The sample was run at 0.2 ml min^−1^ and fractions of 0.5 ml were collected.

### DNA constructs for use as substrates in magnetic-tweezer assays

DNA constructs for magnetic-tweezer experiments of 1.5 kb length were synthesized as described previously^48^.

### DNA constructs for protein expression

Human NIPBL with N-terminal Flag and Halo tags and a C-terminal 10×His tag as a tandem construct with untagged human MAU2 in pLib was described previously^9^. 6×His-Halo-EcoRI^E111Q^ and 6×His-tetR-Halo in pLib were described previously^44^. 10×His-CTCF-Halo-Flag was inserted into pLib by combining the human CTCF ORF and the Halo-tag ORF using Gibson assembly. A C-terminal Flag-tag sequence was introduced as a 5′ overhang in the reverse primer used for Halo-tag ORF amplification. To generate 10×His-CTCF-Halo-Avi-Flag, the 10×His-CTCF-Halo-Flag vector backbone was amplified around the end of the Halo-tag sequence, at which position an Avi-tag was introduced using Gibson assembly.

### Generation of a radioactively labelled dsDNA probe for EMSA

dsDNA fragments (100 bp) containing WT or scrambled versions of the HighOc1 CTCF-binding site^51^ (WT, TCAGAGTGGCGGCCAGCAGGGGGCGCCCTTGCCAGA) were prepared by overlap-extension PCR: two ssDNA oligos with partially overlapping sequences were used in a PCR reaction catalysed by Phusion Hot Start DNA Polymerase (NEB, M0535S) and purified using the PureLink PCR Purification Kit (Invitrogen, K3110002). A total of 1 pmol of dsDNA probe was subsequently incubated with 0.5 µl [γ-^32^P]ATP (3,000 Ci mmol^−1^, 10 mCi ml^−1^; Hartmann Analytic, SCP-301) and T4 polynucleotide kinase (NEB, M0201S) in a 20 µl reaction at 37 °C for 1 h. T4 polynucleotide kinase was subsequently heat-inactivated by incubating the reaction at 65 °C for 10 min.

### Generation of a methylated dsDNA probe for EMSA

A 100 bp dsDNA fragment containing the HighOc1 CTCF-binding site described above^51^ was methylated in vitro using M.SssI CpG methyltransferase (NEB, M0226S) according to the manufacturer’s protocol. To increase methylation efficiency, four rounds of methylation, each followed by DNA purification using the PureLink PCR Purification Kit (Invitrogen, K3110002), were performed. The methylation efficiency was assessed by incubating 300 ng of purified methylated DNA with 1 µl of the methylation-sensitive restriction enzyme EaeI (NEB, R0508S) in a 20 μl reaction containing 1× CutSmart buffer (NEB) at 37 °C for 1 h. The reaction products were resolved by electrophoresis on a 0.8% agarose gel and ethidium bromide staining was detected using the BioRad ChemiDoc Imaging System. The final dsDNA fragment used as unlabelled, methylated competitor in Fig. 1b was methylated with about 80% efficiency.

### Generation of CTCF–Halo–Flag HeLa Kyoto cell line

HeLa Kyoto cells (RRID: CVCL_1922), a gift from S. Narumiya, were cultured as described previously^3^. HeLa Kyoto cells were authenticated by STR fingerprinting and tested negative for mycoplasma contamination. The CTCF-Halo-Flag HeLa Kyoto cell line was generated by homology-directed repair using CRISPR Cas9 (D10A) paired nickase^53^. A donor plasmid comprising CTCF homology arms (719 bp and 459 bp on either side of the coding sequence stop site) and Halo-Flag were cloned into plasmid pJet1.2. Cas9 guide RNA sequences were identified using an online tool (https://crispr.mit.edu; gRNA1: CACCGCAGCATGATGGACCGGTGA; gRNA2: CACCGGAGGATCATCTCGGGCGTG) and inserted into plasmid pX335 (a gift from F. Zhang, Addgene, 42335). HeLa Kyoto cells were transfected with donor Cas9 nickase plasmids using Lipofectamine 2000 (Invitrogen, 11668019). Then, 7 days later, cells were labelled with Halotag TMR ligand (Promega, G8251) and sorted by flow cytometry (Supplementary Fig. 2). The clonal cell line was selected after verification of homozygous Halo-Flag insertion by PCR amplification of genomic DNA, immunoblotting and inspection by microscopy.

### Protein expression and purification

Baculoviruses for protein expression in Sf9 insect cells (Thermo Fisher Scientific) were generated as described previously^54^. Expression cultures were incubated at 27 °C for 48–60 h after infection. Cells were centrifuged, washed in PBS, frozen in liquid nitrogen and stored at −80 °C.

#### Purification of recombinant CTCF protein

Baculovirus-infected cell pellets from cultures supplemented with 0.1 mM ZnCl_2_ were lysed by Dounce homogenization and resuspended in CTCF lysis buffer (35 mM NaH_2_PO_4_/Na_2_HPO_4_ pH 7.4, 350 mM NaCl, 0.1 mM ZnCl_2_, 5% glycerol, 0.05% Tween-20 and 5 mM imidazole) supplemented with 1 mM PMSF, EDTA-free cOmplete tablet (1 per 50 ml) (Roche, 11873580001), 1 mM DTT and 0.001 U µl^−1^ benzonase. The lysate was cleared by centrifugation at 18,000*g* for 1 h at 4 °C. The soluble fraction was incubated with NiNTA agarose (Qiagen, 30230) for 1 h at 4 °C and washed with CTCF buffer (35 mM NaH_2_PO_4_/Na_2_HPO_4_ pH 7.4, 150 mM NaCl, 0.1 mM ZnCl_2_, 5% glycerol) supplemented with 1 mM DTT and 35 mM imidazole. For the final wash step, DTT was omitted from the wash buffer. Protein was eluted with CTCF buffer supplemented with 300 mM imidazole. The eluate was subsequently concentrated approximately twofold using a Sartorius Vivaspin 50 kDa MWCO concentrator (Sartorius, VS2031) and incubated with Anti-FLAG M2 Affinity Gel (Sigma-Aldrich, A2220) for 90 min at 4 °C. The resin was washed with CTCF buffer and incubated with Halotag TMR ligand (Promega, G8252) or Halotag Alexa660 ligand (Promega, G8472) for 15 min at room temperature. After extensive washing with CTCF buffer, the labelled protein was eluted in CTCF buffer supplemented with 0.5 mg ml^−1^ 3×Flag peptide. The eluate was supplemented with 1 mM DTT, concentrated two- to fourfold using the Sartorius Vivaspin 50 kDa MWCO concentrator, flash-frozen and stored at −80 °C.

#### HeLa CTCF–Halo–Flag purification

HeLa CTCF–Halo–Flag protein was purified as described for SCC1–Halo–Flag^9^, except 20 mM Tris pH 7.5 was used in all of the CTCF purification buffers instead of 25 mM NaH_2_PO_4_/Na_2_HPO_4_ pH 7.5, and 0.1 mM ZnCl_2_ was included in all of the purification buffers except for the Flag elution buffer. HeLa CTCF was labelled with JF646-HaloTag ligand. JF646-HaloTag ligand was prepared as described previously^9^.

#### Recombinant cohesin, HeLa cohesin, NIPBL–MAU2 and EcoRI(E111Q) protein purification

Recombinant cohesin, HeLa SCC1–Halo–Flag cohesin and recombinant NIPBL–MAU2 were purified as described previously^9^. EcoRI(E111Q)–Halo and TetR–Halo were purified as described previously^44^.

### EMSA

For the competition EMSA assay, 60 fmol of recombinant CTCF was mixed with 1 µg poly(dI-dC) (Thermo Fisher Scientific, 20148E) in a 20 µl reaction containing 35 mM Tris pH 7.9, 50 mM KCl, 50 mM NaCl, 5 mM MgCl_2_, 0.1 mM ZnCl_2_, 5% glycerol, 1 mM DTT and 50 ng µl^−1^ BSA at room temperature for 20 min. Subsequently, 21 fmol of [γ-^32^P]ATP-labelled (Hartmann Analytic, SCP-501) dsDNA probe was added in the presence of 100× unlabelled competitors (dI-dC; WT; scrambled or methylated CTCF oligo), and the reaction was incubated at room temperature for an additional 10 min. The binding reactions were loaded onto prerun (1 h, 100 V, 10 mA, ice-cold water bath, 0.5× TBE running buffer) 4% non-denaturing acrylamide gel and the samples were resolved for 1 h under the same conditions as the prerun. The gel was exposed to a storage phosphor screen overnight and analysed using a Typhoon Scanner (GE Healthcare). Images shown are representative of two independent experiments.

### Recombinant CTCF single-molecule imaging characterization

#### CTCF flow-in, washing and imaging

Flow cells were incubated with Avidin DN (Vector Laboratories, A3100) and DNA as described previously^9^, except that pPlat containing a single HighOc1 CTCF-binding site was used instead of λ-DNA. Flow cells were washed with 400 µl WB buffer (20 mM Tris pH  7.5, 50 mM KCl, 5 mM EDTA) supplemented with 0.1 mg ml^−1^ BSA and 10 nM Sytox Green (Thermo Fisher Scientific, S7020) or Sytox Orange (Thermo Fisher Scientific, S11368) at 50 µl min^−1^. A total of 100 µl recombinant CTCF–Halo (labelled with TMR in experiments shown in Fig. 1 and in Extended Data Figs. 1a–c,f,g,j and 2; or labelled with Alexa 660 in experiments shown in Extended Data Fig. 1d,e) was then introduced into the flow chamber at 2.5 nM final concentration in CL100 buffer (35 mM Tris pH 7.5, 100 mM KCl, 5 mM MgCl_2_, 5% glycerol, 0.005% Tween-20, 0.1 mg ml^−1^ BSA, 1 mM TCEP) at 30 µl min^−1^ and subsequently incubated for 4 min without buffer flow. Flow cells were then washed with CL150 buffer (CL100 buffer supplemented with 50 mM KCl) at a rate of 50 µl min^−1^ to remove non-specifically bound CTCF molecules.

To determine the orientation of DNA molecules after image acquisition, TMR labelled EcoRI(E111Q)–Halo or TetR–Halo was flowed into the flow cells at 2 nM or 5 nM final concentration, respectively, in EcoRI buffer (20 mM Tris pH 7.5, 150 mM KCl, 0.1 mg ml^−1^ BSA) supplemented with 10 nM Sytox Green at 30 µl min^−1^, incubated for 4 min and washed with 200 µl of EcoRI buffer.

All recombinant CTCF single-molecule imaging characterization and cohesin diffusion assay experiments were performed at room temperature. Unless stated otherwise, time-lapse microscopy images were acquired at 4 s intervals using the Zeiss TIRF 3 Axio Observer set-up and 488 nm, 561 nm and 639 nm lasers^44^. A protocatechuic acid/protocatechuate-3,4-dioxygenase/trolox oxygen scavenger system (final concentration 10 nM protocatechuate-3,4-dioxygenase, 2.5 mM protocatechuic acid and 2 mM trolox); was added to all buffers used during data acquisition.

#### Imaging the kinetics of recombinant CTCF association with DNA

To image the kinetics of CTCF association with DNA (Fig. 1c,d and Extended Data Fig. 1a), 0.5 nM TMR-labelled CTCF–Halo was introduced into flow cells in CL100 buffer at 30 µl min^−1^. For the experiments shown in Fig. 1d and Extended Data Fig. 1a, images were acquired at 3.12 s intervals. For measurements of CTCF residence time on DNA (Fig. 1c and Extended Data Fig. 1f,g) images were acquired at 10.15 s intervals.

#### Positional analysis of recombinant CTCF on DNA

The position of recombinant CTCF on DNA was analysed in Fiji. EcoRI or TetR mediated end-labelling was used to unambiguously assign the orientation of DNA strands tethered to the surface. The distance between the centre of the mass of fluorescence intensity signal marking the DNA end and the fluorescence signal of protein was measured, and the ratio between the measured distance and the total length of the DNA molecule was calculated as a position along the DNA in bp. Single-molecule tracking of the CTCF position was performed using the custom Fiji macro KymoAnalysis_2.1.ijm.

#### CTCF diffusion coefficient analysis

Single-molecule tracking of the CTCF position was performed using the custom Fiji macro KymoAnalysis_2.1.ijm. Spatial positions along the DNA molecule versus time for individual molecules were converted to base pairs by multiplying the positions in micrometres by the average number of base pairs per micrometre, that is, with the factor (26,123 bp)/*R*, where *R* denotes the end-to-end length of the DNA molecule containing 26,123 bp. The MSD was calculated for individual traces and a linear regression in the form MSD(*τ*) = *Dτ* + *o*  was applied to the first ten timepoints (corresponding to a maximum time lag of 31.2 s). Here, *D* denotes the diffusion coefficient, *τ* is the time lag and *o* is an offset to correct for a finite localization uncertainty. Larger time lags were not considered for the regression to exclude artificial flattening of the MSD curves by reaching the DNA ends.

#### Recombinant CTCF photobleaching analysis

To quantify the number of recombinant Alexa 660 (A660)-labelled CTCF molecules bound at a CTCF DNA-binding site, A660 signals on DNA were identified in laser-profile-corrected images, subtracted from the local background, averaged over ten frames and plotted in Extended Data Fig. 1e.

#### Determining the residence time of recombinant CTCF on DNA

To control for fluorophore bleaching in the CTCF in vitro residence-time experiments, the dwell time of ‘on-DNA’ CTCF–HaloTMR molecules (*n* = 140) and ‘on-glass’ CTCF–HaloTMR–Avi–biotin molecules (*n* = 142) (the latter coupled to the biotin-PEGylated glass surface through Avidin DN) was determined by imaging populations of these molecules in the same microfluidic flow cell. We then performed a regression of the fluorescence lifetime distribution to an exponential function on the on-glass population to compute the photobleaching half-life, which was determined to be *T*_1/2_on-glass_ = 77.3 min. The ‘on-DNA’ dataset was best described by a two-exponential decay fit with a fixed percentage of events (97 out of 140, 69%) that displayed rapid unbinding, which were attributed to non-specific DNA-binding events based on their position along the DNA molecule. This resulted in residence times of *T*_1/2_fast_on-DNA_ = 1.2 min and *T*_1/2_slow_on-DNA_ = 29.2 min, corresponding to non-specific and CTCF site-specific DNA-binding events.

Neither single-exponential nor two- or three-exponential fits in which one of the components was fixed to *T*_1/2_on-glass_ was suitable to describe the observed data. On the basis of this and the finding that *T*_1/2_slow_on-DNA_ was ~2.7× shorter than *T*_1/2_on-glass_ (29.2 min and 77.3 min, respectively), we concluded that the off-rate of CTCF on-DNA was significantly faster than the fluorophore bleaching rate and therefore the observed on-DNA dwell time of CTCF was not significantly limited by fluorophore bleaching.

### HeLa CTCF single-molecule imaging characterization

#### CTCF flow-in, washing and imaging

Flow cells^44^ were incubated with 1 mg ml^−1^ Avidin DN (Vector Laboratories) for 15 min and washed extensively with DNA buffer (20 mM Tris pH 7.5, 150 mM NaCl, 0.25 mg ml^−1^ BSA (Thermo Fisher Scientific, AM2616)). A total of 150 µl of 31.8 kb DNA containing a single CTCF site and biotinylated ends was introduced into flow cells at around 20 pM final concentration at 50 µl min^−1^ in DNA buffer supplemented with 20 nM Sytox Orange (Thermo Fisher Scientific, S11368). Flow cells were washed with 400 µl of wash buffer 2 (50 mM Tris pH 7.5, 50 mM NaCl, 2.5 mM MgCl_2_, 0.25 mg ml^−1^ BSA, 0.05% Tween-20, 20 nM Sytox Orange) at 100 µl min^−1^, followed by 100 µl of imaging buffer (50 mM Tris pH 7.5, 50 mM NaCl, 2.5 mM MgCl_2_, 0.25 mg ml^−1^ BSA, 0.05% Tween-20, 0.2 mg ml^−1^ glucose oxidase (Sigma-Aldrich, G2133), 35 mg ml^−1^ catalase (Sigma-Aldrich, C-40), 9 mg ml^−1^ *b*-d-glucose, 2 mM trolox (Cayman Chemical, 10011659)) and 5 mM ATP (Jena Biosciences, NU- 1010-SOL)) supplemented with 20 nM Sytox Orange at 100 µl min^−1^. Stock solutions of glucose oxidase (20 mg ml^−1^), catalase (3.5 mg ml^−1^) and glucose (450 mg ml^−1^) were prepared as described previously^55^. JF646-labelled HeLa CTCF was then introduced into the flow chamber at a final concentration of 0.5 nM in 100 µl imaging buffer supplemented with 20 nM Sytox Orange at 30 µl min^−1^. Non-specifically bound CTCF was removed by washing three times with 100 µl imaging buffer supplemented with 220 nM Sytox Orange at 100 µl min^−1^.

All HeLa CTCF single-molecule characterization and loop-extrusion experiments were performed at 37 °C. Time-lapse microscopy images were acquired using the Zeiss Elyra 7 with Lattice SIM^2^ equipped with 561 nm and 639 nm lasers, two PCO Edge 4.2 sCMOS cameras and a ×63/1.46 NA Alpha Plan-Apochromat oil objective. Images with an exposure time of 100 ms were acquired sequentially for each channel at 0.4 s intervals in HILO mode.

#### HeLa CTCF photobleaching analysis

To quantify the number of HeLa JF646-labelled CTCF molecules bound at a CTCF DNA-binding site, JF646 signals on DNA were identified in laser-profile-corrected images, subtracted from the local background and averaged over all frames before a bleaching event and plotted in Extended Data Fig. 3e. The number of bleaching steps per molecule was determined manually and indicated on Extended Data Fig. 3e. The fluorescence intensity of molecules bound at a CTCF DNA-binding site that bleached in a single step was 2.2 ± 0.6 (mean ± s.d.).

#### HeLa CTCF positional analysis

The position of HeLa CTCF on DNA was analysed as described in the ‘Determination of DNA loop size and position of single molecules’ section (Supplementary Note).

### Cohesin diffusion assay and image analysis

Cohesin diffusion assays were performed essentially as described previously^44^. CTCF was introduced into flow cells at 2 nM final concentration and incubated for 4 min as described in the ‘Recombinant CTCF single-molecule imaging characterization’ section above. Flow cells were then washed with CL150* buffer (35 mM Tris pH 7.5, 75 mM NaCl, 75 mM KCl, 1 mM MgCl_2_, 10% glycerol, 0.003% Tween-20 and 0.1 mg ml^−1^ BSA). Cohesin and NIPBL–MAU2 were introduced into flow cells at 0.8–2 nM and 2 nM, respectively, in 100 µl of CL100* buffer (35 mM Tris, pH 7.5, 50 mM NaCl, 50 mM KCl, 1 mM MgCl_2_, 10% glycerol, 0.003% Tween-20 and 0.1 mg ml^−1^ BSA) at 30 µl min^−1^. Flow cells were incubated for a further 4 min without buffer flow and then washed with CL250* buffer (35 mM Tris pH 7.5, 125 mM NaCl, 125 mM KCl, 1 mM MgCl_2_, 10% glycerol, 0.003% Tween-20 and 0.1 mg ml^−1^ BSA). Cohesin and CTCF imaging was then performed in the absence of buffer flow for 160 s at 4 s per frame intervals. Image acquisition was repeated for 3–5 fields of view. DNA orientation was determined by flowing in Sytox Green and EcoRI(E111Q)–Halo or TetR–Halo as described in the ‘Recombinant CTCF single-molecule imaging characterization’ section above. Biotin-conjugated quantum dots QD705 (Invitrogen, Q101163MP) or CTCF–Halo–Avi–biotin were used as fiducial markers.

CTCF–cohesin channels were aligned with TetR/EcoRI(E111Q)–DNA channels using the custom-written Fiji macro Movement_analysis_macro_Kymo_10c_3Ch.ijm. Each DNA molecule containing diffusing cohesin was manually examined for the presence of a single CTCF signal positioned at the regions in which the CTCF-binding site was introduced. DNA molecules containing multiple or non-specifically bound CTCF molecules were excluded from the analysis. The number of diffusing cohesin foci on the selected DNA molecules was determined and DNA molecules containing more than four mobile cohesin foci were excluded from the analysis. Cohesin behaviour on DNA was then analysed and classified as follows. (1) Cohesin diffusion blocked: (i) cohesin diffuses freely along the DNA and reaches CTCF roadblock, bounces back but does not go past the roadblock during the time of imaging; (ii) cohesin diffuses freely along the DNA, reaches CTCF and becomes immobilized; (iii) two or more cohesin molecules blocked by CTCF. (2) Cohesin passes CTCF in one direction: cohesin passes CTCF during imaging and diffuses back towards CTCF but does not pass back to the other side. (3) Cohesin passes CTCF multiple times.

DNAs with the following events were also excluded from analysis: (1) cohesin diffusing or co-localizing with CTCF. (2) Cohesin failing to encounter CTCF. (3) Cohesin blocked by a high fluorescence intensity CTCF signal, presumably a multimer. (4) Cohesin or CTCF bleaches during image acquisition.

### Loop-extrusion assay

Perpendicular flow loop-extrusion assays were performed essentially as described previously^9,55^. Flow cells were incubated with 1 mg ml^−1^ Avidin DN (Vector Laboratories) for 15 min and washed extensively with DNA buffer (20 mM Tris pH 7.5, 150 mM NaCl, 0.25 mg ml^−1^ BSA (Thermo Fisher Scientific, AM2616)). A total of 40 µl of 31.8 kb DNA containing a single CTCF site and biotinylated ends was introduced into flow cells at about 3 pM final concentration at 15 µl min^−1^ in DNA buffer supplemented with 20 nM Sytox Orange (Thermo Fisher Scientific, S11368). The flow cells were washed with 20 µl of wash buffer 1 (50 mM Tris pH 7.5, 200 mM NaCl, 1 mM MgCl_2_, 5% glycerol, 1 mM DTT, 0.25 mg ml^−1^ BSA, 20 nM Sytox Orange) at 5 µl min^−1^. Flow was then switched to perpendicular mode and a further 350 µl of wash buffer 1 was introduced at 100 µl min^−1^. A total of 400 µl of wash buffer 2 (50 mM Tris pH 7.5, 50 mM NaCl, 2.5 mM MgCl_2_, 0.25 mg ml^−1^ BSA, 0.05% Tween-20, 20 nM Sytox Orange) was then introduced at 100 µl min^−1^, followed by 100 µl of imaging buffer (50 mM Tris pH 7.5, 50 mM NaCl, 2.5 mM MgCl_2_, 0.25 mg ml^−1^ BSA, 0.05% Tween-20, 0.2 mg ml^−1^ glucose oxidase (Sigma-Aldrich, G2133), 35 mg ml^−1^ catalase (Sigma-Aldrich, C-40), 9 mg ml^−1^ *b*-d-glucose, 2 mM trolox (Cayman Chemical, 10011659)) and 5 mM ATP (Jena Biosciences, NU-1010-SOL)) supplemented with 20 nM Sytox Orange at 100 µl min^−1^. JF646-labelled CTCF was then introduced into the flow chamber at 0.5 nM final concentration in 100 µl imaging buffer supplemented with 20 nM Sytox Orange at 30 µl min^−1^. Non-specifically bound CTCF was removed by washing three times with 100 µl imaging buffer supplemented with 220 nM Sytox Orange at 100 µl min^−1^. HeLa cohesin and recombinant NIPBL–MAU2 were then introduced into the flow chamber at 0.5 nM and 3.54 nM, respectively, in 250 µl imaging buffer supplemented with 220 nM Sytox Orange at 30 µl min^−1^.

For loop-extrusion assays in the absence of buffer flow, flow cells were incubated with Avidin DN and washed with DNA buffer as described above. DNA was introduced at 15–25 µl min^−1^ to vary the DNA tension. Flow cells were then washed and incubated as above without switching to perpendicular mode.

### dCas9 binding to DNA

crRNA sequences were chosen at around one-third of the DNA length and, at each end, two sequences were used for efficient binding of the dCas9–gRNA complex per DNA. If located at the same ends, crRNA sequences were spaced at least 2 kb apart to allow discrimination (additionally to bleaching curves) of occasional binding of two dCas9–gRNA complexes per DNA end. Binding sequences were chosen using CRISPOR (http://crispor.tefor.net/crispor.py; PAM indicated in bold): seq7932, ACTGGACTGCGACCGGGCAG**GGG**; seq11802, CGCGGTGGAGGCAGACGTGG**CGG**; seq18967, CTGGTTATGCAGGTCGTAGT**GGG**; and seq21005, GGCATACAAATATTCCATGA**AGG**.

gRNA was obtained by annealing a mixture of universal 67-mer Alt-R CRISPR–Cas9 ATTO550-labelled tracrRNA and crRNA (IDT) matching the binding sites at 95 °C for 2.5 min and slow cooling to 5 °C in steps of 5 °C for 2.5 min each. To couple gRNA to dCas9, 200 nM dCas9 (NEB, NEBM0652T) was mixed with 2 µM gRNA on ice in NEBuffer3.1, incubated at 37 °C for 10 min and placed on ice again.

To bind the dCas9–gRNA complex to DNA, DNA constructs of 31.8 kb length were used to facilitate measurements at a similar end-to-end length and force regime as for the CTCF experiments. DNA was bound to the pegylated glass surface and unbound DNA was washed off with 100 µl imaging buffer. Then, 1 nM dCas9–gRNA was flushed into the flow cell and incubated for 5 min. Non-specifically bound dCas9–gRNA was removed by flushing with 100 µl imaging buffer supplemented with 1 mg ml^−1^ heparin. Heparin was removed by washing with 100 µl imaging buffer. This typically left one to two dCas9–gRNA complexes per DNA. Loop-extrusion experiments were then performed as described above with 30 pM cohesin and 75 pM NIPBL–MAU2. DNA was visualized by staining with 25 nM Sytox Green and exciting with a 488 nm laser. gRNA–ATTO550 was excited by 561 nm laser light in an alternating excitation scheme using a ×60 oil-immersion, 1.49 NA CFI APO TIRF (Nikon) objective. Emission was collected on a Photometrics Prime BSI sCMOS camera using continuous imaging and an exposure time of 100 ms per frame.

### Magnetic-tweezer experiments

The magnetic-tweezer instrument and experiments were conducted essentially as described previously^48^ with minor modifications. The instrument consisted of a pair of vertically aligned (1 mm apart) permanent neodymium-iron-boron magnets (Webcraft) that were was used to generate the magnetic field^56^. The magnet pair was placed on a motorized stage (translation: Physik Instrumente, M-126.PD2; rotation: Physik Instrumente, C-150.PD) and the light of a red LED (*λ* = 630 nm) was allowed to pass the magnet pair gap to illuminate the sample. Transmission was collected by a ×50 oil-immersion objective (CFI Plan 50XH, Achromat; NA = 0.9, Nikon), and the bead diffraction patterns were recorded with a four-megapixel CMOS camera (Falcon, 4M60; Teledyne Dalsa) at 50 Hz. The real-time tracking of the magnetic bead movement in all three dimensions was conducted using LabView 2011-based (National Instruments) control software described and published previously^57,58^. Surface-adhered 1.5 µm polystyrene reference beads (PolySciences) were used as a reference to correct for instrumental drift occurring during measurements. In total, 100–200 beads could be tracked simultaneously in one field of view with a spatial resolution of around 2 nm for the 1.5-kb-long dsDNA tethers^48^.

The flow cell and DNA tethering were prepared as described previously^48^. In brief, the reference beads were diluted 1:1,500 in PBS buffer (pH 7.4; Sigma-Aldrich) and then adhered (~5 min) to the cover glass surface of the flow cell. After removal of non-adhered beads by washing with PBS, sheep digoxigenin antibodies (Roche) at a concentration of 0.1 mg ml^−1^ were incubated in the flow cell for 1 h, after a 500 µl wash with PBS and 2 h incubation with 10 mg ml^−1^ BSA (New England Biolabs, UK) diluted in PBS (pH 7.4) buffer. After washing with 500 µl PBS buffer, 1 pM of the 1.5 kb linear dsDNA construct was incubated in PBS buffer for 20 min in the flow cell. After washing again with 500 µl PBS buffer, Streptavidin-coated superparamagnetic beads (DynaBeads MyOne, LifeTechnologies; diluted 1:400 in PBS) with a diameter of 1 µm were added resulting in the attachment of the beads to the surface-tethered dsDNA constructs after around 5 min; unbound beads were washed out afterwards with PBS.

Before the cohesin loop-extrusion experiments, the quality of tethered dsDNA constructs was assessed by applying a combination of zero and high force (8 pN), and 30 rotations in each direction at high force. Only tethers with singly bound dsDNA and correct DNA end-to-end lengths were used for the subsequent single-molecule experiments. After washing the flow cell with cohesin reaction buffer (40 mM Tris-HCl pH 7.5, 50 mM NaCl, 2.5 mM MgCl_2_, 1 mM DTT, 0.25 mg ml^−1^ BSA, 0.05% Tween-20), 0.1 nM cohesin and 0.25 nM NIPBL–MAU2 were introduced in cohesin buffer supplemented with 2 mM ATP to stretched dsDNA tethers at high force (8 pN). For force-titration experiments (Extended Data Fig. 7), the force was lowered in individual experiments to 1, 0.8, 0.6, 0.4, 0.3, 0.2 and 0.1 pN, and maintained for 10 min. All magnetic-tweezer experiments were performed at room temperature.

The *Z*-bead position over time was extracted using custom-written scripts in IGOR Pro (v.6.37, Wavemetrics), as previously described^48,59^ and a custom-written automated step detection algorithm (MATLAB, MathWorks) was applied to the individual traces as described previously^48,60^ to extract individual loop-extrusion step sizes. Step sizes measured under the same conditions from different traces and experiments were pooled and converted into base pairs^48^ to construct the distribution of cohesin step sizes in dependence of force (Extended Data Fig. 7c,d).

### Simulating the encounter probability of cohesin and CTCF, given force-dependent cohesin step sizes

A 10 kb stretch of DNA was simulated on which CTCF was assumed to be positioned 7 kb from one end. The cohesin-binding site was uniformly sampled along the DNA length. For each force value, step sizes were sampled from the empirically obtained distribution as measured by magnetic-tweezer experiments. The simulations were repeated 500 times for every force value and events in which cohesin came within 50 bp of CTCF were counted as encounters, which constitutes a conservative threshold for the interaction distance between cohesin and CTCF.

### Statistical analysis and reproducibility

Statistical analysis was performed using GraphPad Prism (v.9.4.1) or Python (v.3.7.7) using scipy (v.1.5.2)^61^, numpy (v.1.21.6), trackpy (v.0.4.2)^62^ and statsmodels (v.0.12.2). No statistical methods were used to determine sample size. Experiments were not randomized and the investigators were not blinded to allocation. Figures were assembled using Adobe Illustrator 27.2. All of the experiments were performed at least twice with consistent results. The experiments shown in Fig. 1a,b and Extended Data Figs. 1h,i and 3a,f were performed twice with consistent results. The number of replicates for the experiments shown in Figs. 1g,h, 2e,g and 3c–e and Extended Data Figs. 5b,g,h, 7c, 9a,b,g–l and 10 is listed in the respective figure legends.

### Reporting summary

Further information on research design is available in the Nature Portfolio Reporting Summary linked to this article.

## Online content

Any methods, additional references, Nature Portfolio reporting summaries, source data, extended data, supplementary information, acknowledgements, peer review information; details of author contributions and competing interests; and statements of data and code availability are available at 10.1038/s41586-023-05961-5.

## Supplementary information

Supplementary Information Supplementary Note, Supplementary Tables 1 and 2 and Supplementary Figs. 1 and 2.

Reporting Summary

Supplementary Video 1 Cohesin-mediated DNA loop extrusion blocked after encounter of CTCF from its N-terminal side. The example is the same as shown in Fig. 2c. The raw image series and the resulting kymographs of DNA and CTCF are shown. The images were median filtered with a 5 pixel window. The loop size as well as loop and CTCF positions were quantified as described in the Methods. The left cartoon illustrates the position of CTCF (the tip of the arrow represents its N-terminal side), the loop and the size of the loop. Note that the proportions are only approximately to scale and should be viewed as an illustration, rather than as an absolute quantification. The top view mimics the side-view cartoon and illustrates how changes in loop size are visible as changes in the intensity of the fluorescent ‘dot’ moving along DNA. Instead of varying brightness, loop-size variations as represented as varying diameters of the loop ‘dot’.

Supplementary Video 2 Cohesin-mediated DNA loop extrusion can pass CTCF. When approached at ~50 s, CTCF first blocks the extruding cohesin. Cohesin then retracts while its loop slightly shrinks and attempts again to pass CTCF at 66 s. This attempt was successful as CTCF was included in the loop and translocates with it.

Supplementary Video 3 Repeated short CTCF–loop co-localization events. Cohesin approaches CTCF and encounters it at 74 s, which blocks loop extrusion. After dissociation at 82 s, cohesin attempts to pass CTCF again at 105 s, 139 s and 165 s. The encounter at 175 s results in passing of cohesin and CTCF translocates with the extruded loop.

Supplementary Video 4 Stalling of loop extrusion at CTCF results in a direction switch of loop extrusion. After encounter of cohesin with CTCF at 28 s, the loop continues to grow and the co-localized position of loop and CTCF moves downwards, away from the CTCF–cohesin encounter position. Cohesin and CTCF dissociate at 49 s.

Supplementary Video 5 CTCF-stalled loops shrink in size after dissociating from CTCF. A previously extruded loop is shown. At 30 s, growth of the loop resumes and the loop encounters CTCF shortly after. The loop size and position remain constant while co-localized with CTCF. At 64 s, cohesin and CTCF dissociate and the loop shrinks after which it remains static.

## Data Availability

All data supporting this study are available on reasonable request.  Source data are provided with this paper.

## Source data

Source Data Figs. 1–3 and Source Data Extended Data Figs. 1–3, 5 and 7–10

## Code availability

Custom Python code to analyse and plot blocked fractions, DNA tension, residence times from HILO fluorescence microscopy, as well as IGOR scripts to preprocess magnetic-tweezer data have been deposited at Zenodo (10.5281/zenodo.7409240). MATLAB code to analyse cohesin-mediated steps in magnetic-tweezer data has been previously published and is accessible at Zenodo (10.5281/zenodo.4657659).
