# Supplementary material for: CTCF is a DNA-tension-dependent barrier to cohesin-mediated loop extrusion
Source: Nature. 2023 Apr 19;616(7958):822–7. doi: 10.1038/s41586-023-05961-5 (PMC10132984; doi:10.1038/s41586-023-05961-5)
Supplement: Supplementary file 1 — Supplementary Note, Supplementary Tables 1 and 2 and Supplementary Figs. 1 and 2. [file 41586_2023_5961_MOESM1_ESM.pdf]

nature portfolio

https://doi.org/10.1038/s41586-023-05961-5

Supplementary information

CTCF is a DNA-tension-dependent barrier to cohesin-mediated loop extrusion

In the format provided by the authors and unedited

Supplementary Note

Loop extrusion image analysis

Fluorescence images were cropped to regions of interest spanning single stretched DNA molecules. Cropped images were then further analysed in custom written python-based software as described[46] which allows semi-automated processing and inspection of the data. Images were median-filtered and kymographs were constructed along the long axis of the stretched DNA molecule.

End-to-end length of DNA

The ends of the DNA molecule were determined by a 'peak peeling' algorithm: a time span of the kymograph in which no loop extrusion occurs (usually before flushing in of cohesin) is selected and temporally averaged, which yields an intensity profile of DNA along its long axis. Gaussian peaks of width roughly equal to the full-width-half-maximum (FWHM) of the microscope's point spread function (here 300 nm) were placed at the position of maximum intensity. The height of the Gaussian equals the maximum intensity of the profile. The Gaussian was then subtracted from the intensity profile. The same procedure was iteratively applied on the new profile until less than 10 % of the original area under the intensity profile remained. The location of the outermost peaks corresponded to the ends of the DNA and the difference between the peaks corresponded to the end-to-end length.

Determination of DNA loop size and position of single molecules

Peaks in each frame corresponding to DNA loops or single molecules (CTCF or dCas9, from here on referred to as CTCF) were detected in every frame, if present, using the *scipy.find_peaks* algorithm[61]. Detected spots were then connected using the *trackpy* package[62] which allows for tracking of loop and single molecule positions over time. The intensity of the loop (I_{loop}) is computed as the summation of 7 pixels surrounding the peak position, corrected for the amount of DNA outside of the loop that falls within this window (a more detailed description of this procedure and the associated error is described in the DNA tension measurement error estimation section below). The size of the loop L_{loop} (Extended Data Fig. 10d) is computed by the fraction of intensity attributed to the loop, I_{loop}, in relation to the total intensity along the entire DNA molecule, I, and the DNA length, $L = 31.8\ kbp$,

$$L_{loop} = \frac{I_{loop}}{I} \cdot L.$$

The position of the loop x_{loop} (in bp) is computed relative to one randomly chosen DNA end and thus depends on the integrated DNA intensity between the chosen end to the loop position (the lead DNA I_{lead}; see Extended Data Fig. 10e),

$$x_{loop} = \frac{I_{lead}}{I} \cdot L.$$

The loop-CTCF distance is computed similarly by measuring the DNA intensity between loop and CTCF, $I_{loop-CTCF}$. The integration of the DNA intensity between loop and CTCF position was corrected for half of the loop size since loops appear as Gaussian-shaped foci and overlap the DNA connecting loop and CTCF (more details are described in the DNA tension measurement error estimation section below). The loop-CTCF distance is

$$d_{loop-CTCF} = \frac{I_{loop-CTCF}}{I} \cdot L.$$

The binding position of CTCF was measured relative to the DNA's end points. Given the lower (x_{down}) and upper (x_{up}) DNA end points, the tracked CTCF position in physical space, x_{CTCF}, is converted to its sequence position L_{CTCF} via

$$L_{CTCF} = \frac{x_{CTCF} - x_{down}}{x_{up} - x_{down}} \cdot L,$$

where $(x_{up} - x_{down})$ corresponds to the DNA end-to-end length R .

MSD calculation

From the position of the CTCF over time, the MSD is computed over a moving window, whose length w was roughly adjusted based on the time before and after encounter of the CTCF by cohesin (usually between 21 and 51 frames, corresponding to ~8 s to ~20 s) as

$$\mathrm{MSD}(t) = \frac{1}{w} \sum_{\tau=t-\frac{w}{2}}^{t+\frac{w}{2}} |x_{CTCF}(\tau+1) - x_{CTCF}(\tau)|^2,$$

Force calculation

The DNA tension within the loop is zero in the absence of buffer flow. In the fraction of DNA outside the loop, the tension depends on the end-to-end length of the DNA. The amount of DNA outside the loop, $L_{nonloop}$ is computed by subtracting the loop size from the length of the DNA construct: $L_{nonloop} = L - L_{loop}$. Its contour length $L_{nonloop}^{c}$ is computed as

$$L_{nonloop}^{c} = \alpha L_{nonloop} \cdot 0.342 \frac{\text{nm}}{\text{bp}},$$

accounting for the distance between base pairs of 0.324 nm and α is a correction factor thereof to account for the slightly different contour length of DNA molecules when bound by Sytox Orange [69]. Values of α were measured by generating force-extension curves of 10 kb DNA constructs using Magnetic Tweezers[48] and fit to the worm-like chain (WLC) model which is also used to compute the DNA tension[70] (see Supplementary Table 2).

The relative extension, that is the extension of a DNA molecule relative to its contour length, of the DNA outside of the loop is computed as

$$r = \frac{R}{L_{nonloop}^{c}},$$

where $R = x_{up} - x_{down}$ is the end-to-end length of the tethered DNA molecule (Extended Data Fig. 10d). The empirically determined and well-established force-extension relationship is used to directly convert the relative extension r of a DNA molecule of length $L_{nonloop}$ and contour length $L_{nonloop}^{c}$ tethered to an extension (end-to-end length) R[70]:

$$F = \frac{k_B T}{L_p}\left(\frac{1}{4(1-r)^2} - \frac{1}{4} + \sum_{i=1}^{7} a_i\, r^i\right)$$

where $k_B T = 1.3806503 \cdot 10^{-23} \cdot 310\, K \cdot 10^{-18}\, pN \cdot \mu m$, L_p is the persistence length of DNA at the respective concentration of Sytox Orange (Supplementary Table 2), $a_1 = 1, a_2 = -0.5164228, a_3 = -2.737418, a_4 = 16.07497, a_5 = -38.87607, a_6 = 39.49944, a_7 =$

-14.17718. For an error estimation of these DNA tension measurements, see the DNA tension measurement error estimation section below.

Smoothing of loop size, loop-CTCF distance and MSD traces

Traces of loop size and loop-CTCF distance were filtered by an edge-preserving Chung-Kennedy filter[71] with $N = 4, 8, 16,$ and 32 sampling window lengths, weighting parameter $M = 10$ and sharpness parameter $P = 40$ or a median filter with window size 21 frames.

CTCF-loop co-localization analysis

The duration of CTCF-loop co-localization was identified on smoothed (Chung-Kennedy-filtered) time traces of loop-CTCF distances. Connected stretches at which the loop and CTCF colocalize ($d_{loop-CTCF} = 0$) were identified and components with a gap of only one frame were merged to avoid over-segmenting connected components due to noise in images and erroneous tracking of loop and CTCF positions.

Determination of loop extrusion rates

Loop extrusion rates were determined as described[10]. Due to the short time between cohesin flush-in and encounter with CTCF it was not always possible to measure the loop extrusion rate before CTCF encounter. For this reason, we also compared the LE rate after encounter with CTCF with the LE rate measured in the absence of CTCF (Extended Data Fig. 10).

Determination of loop shrinkage rate, time span and size of slipped loop

Dissociation events were identified by the time point at which the loop-CTCF distance increased above 0 pixels. To avoid confounding effects of loop release with subsequent re-initiation of loop extrusion, the end point of the loop shrinkage period was marked manually at the time point at which the loop stops shrinking. The loop size before the slippage event was computed as an average over the loop size in the 5 frames (2 seconds) before the slippage event (over which the loop size remained constant). The loop size after the slipping event was based on the average of 1-5 frames, depending on if the loop immediately grew again after slipping (then only the last frame in which the

loop still decreases in size can be used) or retained its size (then the loop size was averaged over up to 5 frames). The difference between the loop size before and after slipping corresponds to the fraction of the loop which was lost upon dissociation. The loop shrinkage rate was determined by a linear fit of the loop size between the dissociation time point between loop and cohesin and the time point at which the looped size stopped decreasing.

Fitting of CTCF-loop co-localization time distributions

Distributions of CTCF-loop co-localization times for N- and C-terminal encounters were fit to mono-, bi-exponential and lognormal distributions by a Maximum Likelihood Estimation routine using *scipy.optimize.minimize*[61]. The log-likelihood $\log(L)$ and the number of model parameters k, as well as the number of data points n was used to compute the Bayesian information criterion (BIC) for each of the models and distributions as displayed in Extended Data Fig. 8e: BIC $= k \cdot log(n) - 2L$.

Measuring the stalling force of cohesin

The stalling force of cohesin was measured on DNA molecules without binding protein (i.e. without CTCF, dCas9 or EcoRI) which were stretched to > 50% of its contour length (contour length ~11.6 µm for a DNA length of 31.8 kb in imaging buffer containing 100 nM Sytox Orange). On such stretched molecules, the stalling force is reached before the cohesin reaches one end of the DNA, thus eliminating confounding effects from the DNA ends. For kymographs with multiple extrusion, slipping and direction change events (as e.g. in Extended Data Fig. 4a, b), the highest force value (as long as it did not approach one of the DNA ends) during the acquisition time was considered as the stalling force. Note that, in contrast to the stalling forces measured using the fluorescence single-molecule assay, cohesin-mediated loop extrusion steps measured using Magnetic Tweezers (see below) were still detectable at forces up to 1 pN. At this high force only a single or a few steps were observed per loop extrusion event. Since this would result in loop sizes ≤ 1 kb, they would thus not be visible in the fluorescence assay. Additionally, cohesin molecules may stochastically halt loop extrusion even on DNA with low end-to-end distance and thus at very low tension (see e.g. Extended Data Fig. 4a, timepoint 62 s). Thus, a mixture of cohesin molecules were measured, some of which

stopped extruding due to reaching the stalling force and some stopped for other reasons. Therefore, the stalling force shown in Extended Data Fig. 5b is thus a conservative estimate of the stalling force, but is comparable to encounters with CTCF and dCas9 since these data points were acquired under identical conditions.

Computation of the combinatorial probability of CTCF-CTCF loops

The probability to observe CTCF-CTCF loops with the two respective CTCF sites in a convergent (><), tandem (>> and <<), or divergent (<>) manner, as reported previously from Hi-C data[3,63-65], was obtained from the loop extrusion stalling probability upon encounter with CTCF on its N-terminal (P^N) and C-terminal (P^C) side in the force range 0.04-0.08 pN, as shown in Fig. 2e:

$$P(><) = P^N P^N / A$$

$$P(\gg \vee \ll) = P^N P^C / A$$

$$P(<>) = P^C P^C / A,$$

where A is a normalization constant, i.e. $A = P(><) + P(\gg \vee \ll) + P(<>)$.

DNA tension measurement error estimation

The error σ_F of the DNA tension F was computed via error propagation:

$$\sigma_F^2 = \left(\frac{\partial F}{\partial L_p}\sigma_{L_p}\right)^2 + \left(\frac{\partial F}{\partial T}\sigma_T\right)^2 + \left(\frac{\partial F}{\partial r}\sigma_r\right)^2$$

$$= \left(-F\frac{\sigma_{L_p}}{L_p}\right)^2 + \left(F\frac{\sigma_T}{T}\right)^2 + \left(\frac{k_B T}{L_p}\left(\frac{1}{2(1-r)^3} + \sum_{i=1}^{7} i a_i\, r^{i-1}\right)\sigma_r\right)^2$$

where $\frac{\partial F}{\partial x_i}$ denote partial derivatives of F with respect to the variables x_i. Here, the variables x_i represent the temperature T, persistence length L_p and the relative extension r.

We estimate the contribution on the temperature T, persistence length L_p and the relative extension r separately. Here, we perform a 'worst case' analysis on the resulting error, i.e. we conservatively estimate the uncertainties on the contributing parameters and establish a reasonable upper bound on the resulting error.

Persistence length: Fitting of average force extension curves $\pm$ one standard deviation yields a persistence length of $L_p = 37.1\ nm$ and $\sigma_{L_p} = 2.1\ nm$.

Temperature: The experiments were temperature controlled and we assume a deviation of the set temperature within 1 K, i.e. $\sigma_T = 1\ K$.

Relative extension: The relative extension is the fraction of the DNA end-to-end length R and the contour length of the DNA outside the loop $L^c_{nonloop}$

$$r = \frac{R}{L^c_{nonloop}},$$

where $L^c_{nonloop} = \alpha \cdot 0.342 \frac{nm}{bp} \cdot L_{nonloop}$. Its error is computed as

$$\sigma_r^2 = \left(\frac{\sigma_R}{L^c_{nonloop}}\right)^2 + \left(\frac{\sigma_{L^c_{nonloop}} R}{\left(L^c_{nonloop}\right)^2}\right)^2,$$

where $\sigma_{L^c_{nonloop}} = \alpha \cdot 0.342 \frac{nm}{bp} \sigma_{L_{nonloop}}$.

The uncertainty in end-to-end length σ_R is estimated to be in the order of the PSF width due to convolution of the DNA ends with the PSF. The width of the microscope PSF was determined to be $\sigma_{PSF} = 180 \pm 13\ nm$ (Extended Data Fig. 10g-h) on single emitters. Due to a potential contribution of DNA intensity beyond the tether points due to the flexibility of DNA (Extended Data Fig. 10f; this contribution diminishes after averaging the DNA profile over multiple frames), we conservatively set $\sigma_R = 250\ nm$.

The amount of non-extruded DNA is computed from the total DNA length ($L = 31.8\ kb$) and the intensity of the extruded loop I_{loop} with respect to the total DNA intensity I, i.e. $L_{nonloop} = L\left(1 - \frac{I_{loop}}{I}\right)$. The uncertainty of the DNA amount outside the loop is thus

$$\sigma^2_{L_{nonloop}} = \left(L\frac{\sigma_{I_{loop}}}{I}\right)^2 + \left(-L\frac{I_{loop}\sigma_I}{I^2}\right)^2.$$

The total DNA intensity I is constant throughout the experiment due to exchanging Sytox Orange molecules, making DNA photobleaching for the duration of the experiment negligible. Furthermore, several thousand images are acquired, effectively diminishing the uncertainty of the total DNA intensity I. We thus set $\sigma_I \approx 0$. The uncertainty of the loop intensity can be estimated as follows:

An illustration of the DNA intensity profile along its long axis is shown in Extended Data Fig. 10f. The extruded loop appears as protrusion on top of the DNA intensity stemming from the non-extruded fraction of the DNA construct. To quantify the loop intensity I_{loop}, we first find the position at the maximum intensity, x_{loop}. We then integrate the intensity profile within a 7-pixel window ($w = 7$) around x_{loop}. However, not only DNA within the loop, but also non-extruded DNA contributes to this integral since DNA in the loop and outside cannot be spatially distinguished. We thus correct the integral by measuring the average DNA intensity per pixel $\langle\frac{\partial I}{\partial x}\rangle$ at least two pixels ($\approx 200\ nm$) away from the loop and the DNA ends, i.e. without extruded loop. The loop intensity I_{loop} is thus computed as

$$I_{loop} = \int_{x_{loop}-w/2}^{x_{loop}+w/2} I(x)\, dx - w \langle\frac{\partial I}{\partial x}\rangle\,.$$

The first term describes the integration over a window of size w around the peak maximum position x_{loop} and the second term describes the correction due to the contribution of the DNA intensity from the non-extruded DNA to the integral. The peak is roughly Gaussian-shaped due to the convolution of the loop intensity with the point spread function (PSF) of the microscope. The integral over the peak can be solved explicitly in this case:

$$I_{loop} = \frac{A}{\sqrt{2\pi}\sigma} \int_{x_{loop}-\frac{w}{2}}^{x_{loop}+\frac{w}{2}} e^{-\frac{(x-x_{loop})^2}{2\sigma^2}}\, dx$$

$$= \frac{A}{2}\left(\operatorname{erf}\left(\frac{w}{2\sqrt{2}\sigma}\right) - \operatorname{erf}\left(\frac{-w}{2\sqrt{2}\sigma}\right)\right)$$

A is a normalization constant, erf() is the Error function, and σ is the width of the peak corresponding to the extruded loop. An integral over the entire Gaussian (from $-\infty$ to ∞ instead of $x_{loop} - \frac{w}{2}$ to $x_{loop} + \frac{w}{2}$) yields A. The error by truncating the integral outside the range $[x_{loop} - \frac{w}{2}, x_{loop} + \frac{w}{2}]$ is thus

$$\sigma_{I_{loop}} = I_{loop}\left(1 - \frac{1}{2}\left(\mathrm{erf}\left(\frac{w}{2\sqrt{2}\sigma}\right) - \mathrm{erf}\left(\frac{-w}{2\sqrt{2}\sigma}\right)\right)\right)$$

$\sigma_{I_{loop}}$ is monotonic and increases for larger values of σ. To estimate the width of the Gaussian, we consider a loop of size 10 kb, i.e. $L_{loop} = 10\,kb$, which is close to the maximum loop size in our experiments on the DNA template of $L = 31.8\,kb$ (94% of loop sizes are below 10 kb), yielding the maximum encountered value for the loop peak width σ. The intensity from this signal is convolved with the microscope PSF. An upper bound of the physical dimensions of the unconstrained loop (there is no tension acting on DNA in the loop) can be estimated by the radius of gyration of a circular DNA molecule $R_g = \sqrt{\frac{Nb^2}{12}}$. The quantity $b = 2L_p$ is the Kuhn length and the chain is made of $N = \frac{L_{loop} \cdot 0.342 \frac{nm}{bp}}{b} \approx 49$ segments, yielding $R_g \approx 140\,nm$. For simplicity, we assume that both the PSF and the DNA distribution of the loop are Gaussian-shaped. The convolution of the loop with the PSF is then described by a Gaussian with width $\sigma = \sqrt{R_g^2 + \sigma_{PSF}^2} \approx 230\,nm$, with $\sigma_{PSF} = 180 \pm 13\,nm$ (Extended Data Fig. 10g-h). For a loop size $L_{loop} = 10\,kb$, the relative error of the loop intensity is thus $\sigma_{I_{loop}} \approx 15\%$ and $\sigma_{I_{loop}} \approx 9\%$ for a loop size of 3 kb.

We can express the error of the length of the non-extruded DNA irrespective of the absolute intensity value since intensity and DNA content scale linearly under the assumption of a constant density of Sytox Orange dye molecules along the DNA.

$$\sigma_{L_{nonloop}} = L\frac{\sigma_{I_{loop}}}{I}$$

$$= \mathrm{L}\frac{I_{loop}}{I}\left(1 - \frac{1}{2}\left(\mathrm{erf}\left(\frac{w}{2\sqrt{2}\sigma}\right) - \mathrm{erf}\left(\frac{-w}{2\sqrt{2}\sigma}\right)\right)\right)$$

$$= L_{loop}\left(1 - \frac{1}{2}\left(\mathrm{erf}\left(\frac{w}{2\sqrt{2}\sigma}\right) - \mathrm{erf}\left(\frac{-w}{2\sqrt{2}\sigma}\right)\right)\right)$$

We can thus evaluate the uncertainty of the relative extension, σ_r by using the determined values for σ_R and $\sigma^c_{L_{nonloop}} = \alpha \cdot 0.342\frac{nm}{bp} \cdot \sigma_{\mathrm{L}_{\mathrm{nonloop}}}$.

Illustratively, the absolute and relative error is evaluated for fixed end-to-end length R and fixed loop size L_{loop} in Extended Data Fig. 10i-j. For relevant end-to-end length values and loop sizes, the relative error of the DNA tension measurement is $< 17\%$.

References

1 Davidson, I. F. & Peters, J. M. Genome folding through loop extrusion by SMC complexes. *Nat Rev Mol Cell Biol* **22**, 445-464, doi:10.1038/s41580-021-00349-7 (2021).

2 Nora, E. P. *et al.* Targeted Degradation of CTCF Decouples Local Insulation of Chromosome Domains from Genomic Compartmentalization. *Cell* **169**, 930-944 e922, doi:10.1016/j.cell.2017.05.004 (2017).

3 Wutz, G. *et al.* Topologically associating domains and chromatin loops depend on cohesin and are regulated by CTCF, WAPL, and PDS5 proteins. *EMBO J* **36**, 3573-3599, doi:10.15252/embj.201798004 (2017).

4 Ba, Z. *et al.* CTCF orchestrates long-range cohesin-driven V(D)J recombinational scanning. *Nature* **586**, 305-310, doi:10.1038/s41586-020-2578-0 (2020).

5 Hill, L. *et al.* Wapl repression by Pax5 promotes V gene recombination by Igh loop extrusion. *Nature* **584**, 142-147, doi:10.1038/s41586-020-2454-y (2020).

6 Olbrich, T. *et al.* CTCF is a barrier for 2C-like reprogramming. *Nat Commun* **12**, 4856, doi:10.1038/s41467-021-25072-x (2021).

7 Segueni, J. & Noordermeer, D. CTCF: A misguided jack-of-all-trades in cancer cells. *Comput Struct Biotechnol J* **20**, 2685-2698, doi:10.1016/j.csbj.2022.05.044 (2022).

8 Hansen, A. S. CTCF as a boundary factor for cohesin-mediated loop extrusion: evidence for a multi-step mechanism. *Nucleus* **11**, 132-148, doi:10.1080/19491034.2020.1782024 (2020).

9 Davidson, I. F. *et al.* DNA loop extrusion by human cohesin. *Science* **366**, 1338-1345, doi:10.1126/science.aaz3418 (2019).

10 Ganji, M. *et al.* Real-time imaging of DNA loop extrusion by condensin. *Science* **360**, 102-105, doi:10.1126/science.aar7831 (2018).

11 Golfier, S., Quail, T., Kimura, H. & Brugues, J. Cohesin and condensin extrude DNA loops in a cell-cycle dependent manner. *Elife* **9**, e53885 (2020).

12 Kim, Y., Shi, Z., Zhang, H., Finkelstein, I. J. & Yu, H. Human cohesin compacts DNA by loop extrusion. *Science* **366**, 1345-1349, doi:10.1126/science.aaz4475 (2019).

13 Kong, M. *et al.* Human Condensin I and II Drive Extensive ATP-Dependent Compaction of Nucleosome-Bound DNA. *Mol Cell* **79**, 99-114, doi:10.1016/j.molcel.2020.04.026 (2020).

14 Pradhan, B. *et al.* The Smc5/6 complex is a DNA loop extruding motor. *bioRxiv*, 2022.2005.2013.491800, doi:10.1101/2022.05.13.491800 (2022).

15 Beckwith, K. *et al.* Visualization of loop extrusion by DNA nanoscale tracing in single human cells. *bioRxiv*, 2021.2004.2012.439407, doi:10.1101/2021.04.12.439407 (2022).

16 Flyamer, I. M. *et al.* Single-nucleus Hi-C reveals unique chromatin reorganization at oocyte-to-zygote transition. *Nature* **544**, 110-114, doi:10.1038/nature21711 (2017).

17 Gabriele, M. *et al.* Dynamics of CTCF- and cohesin-mediated chromatin looping revealed by live-cell imaging. *Science* **376**, 496-501, doi:10.1126/science.abn6583 (2022).

18 Dixon, J. R. *et al.* Topological domains in mammalian genomes identified by analysis of chromatin interactions. *Nature* **485**, 376-380, doi:10.1038/nature11082 (2012).

19 Nora, E. P. *et al.* Spatial partitioning of the regulatory landscape of the X-inactivation centre. *Nature* **485**, 381-385, doi:10.1038/nature11049 (2012).

20 Busslinger, G. A. *et al.* Cohesin is positioned in mammalian genomes by transcription, CTCF and Wapl. *Nature* **544**, 503-507, doi:10.1038/nature22063 (2017).

21 Hashimoto, H. *et al.* Structural Basis for the Versatile and Methylation-Dependent Binding of CTCF to DNA. *Mol Cell* **66**, 711-720.e713, doi:10.1016/j.molcel.2017.05.004 (2017).

22 Yin, M. *et al.* Molecular mechanism of directional CTCF recognition of a diverse range of genomic sites. *Cell Res* **27**, 1365-1377, doi:10.1038/cr.2017.131 (2017).

23 Fudenberg, G. *et al.* Formation of Chromosomal Domains by Loop Extrusion. *Cell Rep* **15**, 2038-2049, doi:10.1016/j.celrep.2016.04.085 (2016).

24 Nichols, M. H. & Corces, V. G. A CTCF Code for 3D Genome Architecture. *Cell* **162**, 703-705, doi:10.1016/j.cell.2015.07.053 (2015).

25 Sanborn, A. L. *et al.* Chromatin extrusion explains key features of loop and domain formation in wild-type and engineered genomes. *Proc Natl Acad Sci U S A* **112**, E6456-6465, doi:10.1073/pnas.1518552112 (2015).

26 Li, Y. *et al.* The structural basis for cohesin-CTCF-anchored loops. *Nature* **578**, 472-476, doi:10.1038/s41586-019-1910-z (2020).

27 Nishana, M. *et al.* Defining the relative and combined contribution of CTCF and CTCFL to genomic regulation. *Genome Biol* **21**, 108, doi:10.1186/s13059-020-02024-0 (2020).

28 Nora, E. P. *et al.* Molecular basis of CTCF binding polarity in genome folding. *Nat Commun* **11**, 5612, doi:10.1038/s41467-020-19283-x (2020).

29 Pugacheva, E. M. *et al.* CTCF mediates chromatin looping via N-terminal domain-dependent cohesin retention. *Proc Natl Acad Sci U S A* **117**, 2020-2031, doi:10.1073/pnas.1911708117 (2020).

30 Liu, Y. & Dekker, J. Biochemically distinct cohesin complexes mediate positioned loops between CTCF sites and dynamic loops within chromatin domains. *bioRxiv*, 2021.2008.2024.457555, doi:10.1101/2021.08.24.457555 (2021).

31 Shaltiel, I. A. *et al.* A hold-and-feed mechanism drives directional DNA loop extrusion by condensin. *Science* **376**, 1087-1094, doi:10.1126/science.abm4012 (2022).

32 Vian, L. *et al.* The Energetics and Physiological Impact of Cohesin Extrusion. *Cell* **173**, 1165-1178 e1120, doi:10.1016/j.cell.2018.03.072 (2018).

33 Emerson, D. J. *et al.* Cohesin-mediated loop anchors confine the locations of human replication origins. *Nature* **606**, 812-819, doi:10.1038/s41586-022-04803-0 (2022).

34 Mitter, M. *et al.* Conformation of sister chromatids in the replicated human genome. *Nature* **586**, 139-144, doi:10.1038/s41586-020-2744-4 (2020).

35 Bell, A. C. & Felsenfeld, G. Methylation of a CTCF-dependent boundary controls imprinted expression of the Igf2 gene. *Nature* **405**, 482-485, doi:10.1038/35013100 (2000).

36 Bell, A. C., West, A. G. & Felsenfeld, G. The protein CTCF is required for the enhancer blocking activity of vertebrate insulators. *Cell* **98**, 387-396, doi:10.1016/s0092-8674(00)81967-4 (1999).

37 Agarwal, H., Reisser, M., Wortmann, C. & Gebhardt, J. C. M. Direct Observation of Cell-Cycle-Dependent Interactions between CTCF and Chromatin. *Biophysical Journal* **112**, 2051-2055, doi:https://doi.org/10.1016/j.bpj.2017.04.018 (2017).

38 Hansen, A. S., Amitai, A., Cattoglio, C., Tjian, R. & Darzacq, X. Guided nuclear exploration increases CTCF target search efficiency. *Nature Chemical Biology* **16**, 257-266, doi:10.1038/s41589-019-0422-3 (2020).

39 Hansen, A. S., Pustova, I., Cattoglio, C., Tjian, R. & Darzacq, X. CTCF and cohesin regulate chromatin loop stability with distinct dynamics. *Elife* **6**, e25776 (2017).

40 Kieffer-Kwon, K. R. *et al.* Myc Regulates Chromatin Decompaction and Nuclear Architecture during B Cell Activation. *Mol Cell* **67**, 566-578 e510, doi:10.1016/j.molcel.2017.07.013 (2017).

41 Nakahashi, H. *et al.* A genome-wide map of CTCF multivalency redefines the CTCF code. *Cell Rep* **3**, 1678-1689, doi:10.1016/j.celrep.2013.04.024 (2013).

42 Soochit, W. *et al.* CTCF chromatin residence time controls three-dimensional genome organization, gene expression and DNA methylation in pluripotent cells. *Nat Cell Biol* **23**, 881-893, doi:10.1038/s41556-021-00722-w (2021).

43 Zhang, H. *et al.* CTCF and R-loops are boundaries of cohesin-mediated DNA looping. *bioRxiv*, 2022.2009.2015.508177, doi:10.1101/2022.09.15.508177 (2022).

44 Davidson, I. F. *et al.* Rapid movement and transcriptional re-localization of human cohesin on DNA. *EMBO J* **35**, 2671-2685, doi:10.15252/embj.201695402 (2016).

45 Haering, C. H., Farcas, A. M., Arumugam, P., Metson, J. & Nasmyth, K. The cohesin ring concatenates sister DNA molecules. *Nature* **454**, 297-301, doi:10.1038/nature07098 (2008).

46 Pradhan, B. *et al.* SMC complexes can traverse physical roadblocks bigger than their ring size. *Cell Rep* **41**, 111491, doi:10.1016/j.celrep.2022.111491 (2022).

47 Martinez, S. R. & Miranda, J. L. CTCF terminal segments are unstructured. *Protein Sci* **19**, 1110-1116, doi:10.1002/pro.367 (2010).

48 Ryu, J. K. *et al.* Condensin extrudes DNA loops in steps up to hundreds of base pairs that are generated by ATP binding events. *Nucleic Acids Res* **50**, 820-832, doi:10.1093/nar/gkab1268 (2022).

49 Tišma, M. *et al.* ParB proteins can bypass DNA-bound roadblocks via dimer-dimer recruitment. *Sci Adv* **8**, eabn3299, doi:10.1126/sciadv.abn3299 (2022).

50 Wutz, G. *et al.* ESCO1 and CTCF enable formation of long chromatin loops by protecting cohesin(STAG1) from WAPL. *Elife* **9**, e52091 (2020).

51 Plasschaert, R. N. *et al.* CTCF binding site sequence differences are associated with unique regulatory and functional trends during embryonic stem cell differentiation. *Nucleic Acids Res* **42**, 774-789, doi:10.1093/nar/gkt910 (2014).

52 Gibson, D. G. *et al.* Enzymatic assembly of DNA molecules up to several hundred kilobases. *Nat Methods* **6**, 343-345, doi:10.1038/nmeth.1318 (2009).

53 Ran, F. A. *et al.* Double nicking by RNA-guided CRISPR Cas9 for enhanced genome editing specificity. *Cell* **154**, 1380-1389, doi:10.1016/j.cell.2013.08.021 (2013).

54 Weissmann, F. & Peters, J. M. Expressing Multi-subunit Complexes Using biGBac. *Methods Mol Biol* **1764**, 329-343, doi:10.1007/978-1-4939-7759-8_21 (2018).

55 Bauer, B. W. *et al.* Cohesin mediates DNA loop extrusion by a "swing and clamp" mechanism. *Cell* **184**, 5448-5464.e5422, doi:10.1016/j.cell.2021.09.016 (2021).

56 Lipfert, J., Hao, X. & Dekker, N. H. Quantitative modeling and optimization of magnetic tweezers. *Biophys J* **96**, 5040-5049, doi:10.1016/j.bpj.2009.03.055 (2009).

57 Cnossen, J. P., Dulin, D. & Dekker, N. H. An optimized software framework for real-time, high-throughput tracking of spherical beads. *Rev Sci Instrum* **85**, 103712, doi:10.1063/1.4898178 (2014).

58 De Vlaminck, I. *et al.* Mechanism of homology recognition in DNA recombination from dual-molecule experiments. *Mol Cell* **46**, 616-624, doi:10.1016/j.molcel.2012.03.029 (2012).

59 Janissen, R. *et al.* Global DNA Compaction in Stationary-Phase Bacteria Does Not Affect Transcription. *Cell* **174**, 1188-1199.e1114, doi:10.1016/j.cell.2018.06.049 (2018).

60 Loeff, L., Kerssemakers, J. W. J., Joo, C. & Dekker, C. AutoStepfinder: A fast and automated step detection method for single-molecule analysis. *Patterns (N Y)* **2**, 100256, doi:10.1016/j.patter.2021.100256 (2021).

61 Virtanen, P. *et al.* SciPy 1.0: fundamental algorithms for scientific computing in Python. *Nat Methods* **17**, 261-272, doi:10.1038/s41592-019-0686-2 (2020).

62 Allan, D., Caswell, T., Keim, N., van der Wel, C. M. & Verweij, R. soft-matter/trackpy: Trackpy v0. 5.0. *Genève: Zenodo* (2021).

63 de Wit, E. *et al.* CTCF Binding Polarity Determines Chromatin Looping. *Mol Cell* **60**, 676-684, doi:10.1016/j.molcel.2015.09.023 (2015).

64 Guo, Y. *et al.* CRISPR Inversion of CTCF Sites Alters Genome Topology and Enhancer/Promoter Function. *Cell* **162**, 900-910, doi:10.1016/j.cell.2015.07.038 (2015).

65 Haarhuis, J. H. I. *et al.* The Cohesin Release Factor WAPL Restricts Chromatin Loop Extension. *Cell* **169**, 693-707 e614, doi:10.1016/j.cell.2017.04.013 (2017).

66 Ainavarapu, S. R. *et al.* Contour length and refolding rate of a small protein controlled by engineered disulfide bonds. *Biophys J* **92**, 225-233, doi:10.1529/biophysj.106.091561 (2007).

67 Kuhn, W. Über die Gestalt fadenförmiger Moleküle in Lösungen. *Kolloid-Zeitschrift* **68**, 2-15, doi:10.1007/BF01451681 (1934).

68 Nomidis, S. K., Carlon, E., Gruber, S. & Marko, J. F. DNA tension-modulated translocation and loop extrusion by SMC complexes revealed by molecular dynamics simulations. *Nucleic Acids Res* **50**, 4974-4987, doi:10.1093/nar/gkac268 (2022).

69 Biebricher, A. S. *et al.* The impact of DNA intercalators on DNA and DNA-processing enzymes elucidated through force-dependent binding kinetics. *Nat Commun* **6**, 7304, doi:10.1038/ncomms8304 (2015).

70 Bouchiat, C. *et al.* Estimating the Persistence Length of a Worm-Like Chain Molecule from Force-Extension Measurements. *Biophysical Journal* **76**, 409-413, doi:https://doi.org/10.1016/S0006-3495(99)77207-3 (1999).

71 Chung, S. H. & Kennedy, R. A. Forward-backward non-linear filtering technique for extracting small biological signals from noise. *J Neurosci Methods* **40**, 71-86, doi:10.1016/0165-0270(91)90118-j (1991).

Supplementary Table 1. Cloning scheme: Plasmid (number and name), Cloning reaction, templates used and primers

#64	pBlueScript-GoldenGate			
KLD cloning	Primers	Sequence	Template	
PCR (Q5 NEB E0555L)	JT290	CGACAAGGTCTCGTCCACTAGTTCTAGAGCGG	#61	pBlueScript-noBsaI
	JT291	CGACAAGGTCTCGTCGTATATCGATACCGTCGACCT		
KLD reaction	NEB M0554S			

#61	pBlueScript-noBsaIsite			
KLD cloning				
PCR (Q5 NEB E0555L)	JT288	GGACCCACGCTCACCGGCTC	#18	pBlueScriptII SK+ (stratagene)
	JT289	CGCGGTATCATTGCAGCACTG		
KLD reaction	NEB M0554S			

#66	pGGA-JT292JT293			
Blunt ligation	Primers	Sequence	Template	
PCR (Q5 NEB E0555L)	JT292	CGACAAGGTCTCGACGAAATGCGCGTATGGGGATGG	Lambda	NEB N3011S
	JT293	CGACAAGGTCTCGGGCTCCGTGGGCCAGGTGGT		
PCR (Q5 NEB E0555L)	JT403	CCTGTAGTCTTCTTAATTAAGACGTCAG	pGGAselect	NEB N0309AAVIAL
	JT401	GTACCAAGTCTTCGAATTCGGATC		
Blunt ligation	Enzymes used: T4 PNK (NEB M0201), DPNI (NEB R0176), T4 DNA Ligase (NEB M0202)			

#67	pGGA-JT294JT295			
Blunt ligation	Primers	Sequence	Template	
PCR (Q5 NEB E0555L)	JT294	CGACAAGGTCTCCAGCCGCGACTTACCATGTAT		
	JT295	CGACAAGGTCTCCATACCGCGATGGTTGGAGTTCCA	**Template GeneBlock34 (IDT):**	

CCGCGACTTACCATGTATCTCGTGCGGAACGCTCACGTCTGCTCAGTGGGATGCCGGACATTACCGGACAACTGCTGCGGCACCTCAACTCCGATTTAATGAACGCAATATTCACAAGCAATGCGTGGTGTGCAACCAGCACA
AAAGCGGAAATCTCGTTCCGTATCGCGTCGAACTGATTAGCCGCATCGGGCAGGAAGCAGTAGACGAAATCGAATCAAACCATAACCGCCATCGCTGGACTATCGAAGAGTGCAAGGCGATCAAGGCAGAGTACCAACAG
AAACTCAAAGACCTGCGAAATAGCAGAAGTGAGGCCGCATGACGTTCTCAGTAAAAACCATTCCAGACATGCTCGTTGAAGCATACGGAAATCAGACAGAAGTAGCACGCAGACTGAAATGTAGTCGCGGTACGGTCAGA
AAATACGTTGATGATAAAGACGGGAAAATGCACGCCATCGTCAACGACGTTCTCATGGTTCATCGCGGATGGAGTGAAAGAGATGCGCTATTACGAAAAAATTGATGGCAGCAAATACCGAAATATTTGGGTAGTTGGCGA
TCTGCACGGATGCTACACGAACCTGATGAACAAACTGGATACGATTGGATTCGACAACAAAAAAGACCTGCTTATCTCGGTGGGCGATTTGGTTGATCGTGGTGCAGAGAACGTTGAATGCCTGGAATTAATCACATTCCCCT
GGTTCAGAGCTGTACGTGGAAACCATGAGCAAATGATGATTGATGGCTTATCAGAGCGTGGAAACGTTAATCACTGGCTGCTTAATGGCGGTGGCTGGTTCTTTAATCTCGATTACGACAAAGAAATTCTGGCTAAAGCTCTTG
CCCATAAAGCAGATGAACTTCCGTTAATCATCGAACTGGTGAGCAAAGATAAAAAATATGTTATCTGCCACGCCGATTATCCCTTTGACGTATACGAGTTTGGAAAGCCAGTTGATCATCAGCAGGTAATCTGGAACCGCGAA
CGAATCAGCAACTCACAAAACGGGATCGTGTGGCCACCAGGGGGCGCTAAAAGAAATCAAAGGCGCGGACACGTTCATCTTTGGTCATACGCCAGCAGTGAAACCACTCAAGTTTGCCAACCAAATGTATATCGATACCGG
CGCAGTGTTCTGCGGAAACCTAACATTGATTCAGGTACAGGGAGAAGGCGCATGAGACTCGAAAGCGTAGCTAAATTTCATTCGCCAAAAAGCCCGATGATGAGCGACTCACCACGGGCCACGGCTTCTGACTCTCTTTCCG
GTACTGATGTGATGGCTGCTATGGGGATGGCGCAATCACAAGCCGGATTCGGTATGGCTGCATTCTGCGGTAAGCACGAACTCAGCCAGAACGACAAACAAAAGGCTATCAACTATCTGATGCAATTTGCACACAAGGTATC
GGGGAAATACCGTGGTGTGGCAAAGCTTGAAGGAAATACTAAGGCAAAGGTACTGCAAGTGCTCGCAACATTCGCTTATGCGGATTATTGCCGTAGTGCCGCGACGCCGGGGGCAAGATGCAGAGATTGCCATGGTACAG
GCCGTGCGGTTGATATTGCCAAAACAGAGCTGTGGGGGAGAGTTGTCGAGAAAGAGTGCGGAAGATGCAAAGGCGTCGGCTATTCAAGGATGCCAGCAAGCGCAGCATATCGCGCTGTGACGATGCTAATCCCAAACCTT
ACCCAACCCACCTGGTCACGCACTGTTAAGCCGCTGTATGACGCTCTGGTGGTGCAATGCCACAAAGAAGAGTCAATCGCAGACAACATTTTGAATGCGGTCACACGTTAGCAGCATGATTGCCACGGATGGCAACATATTA
ACGGCATGATATTGACTTATTGAATAAAATTGGGTAAATTTGACTCAACGATGGGTTAATTCGCTCGTTGTGGTAGTGAGATGAAAAGAGGCGGCGCTTACTACCGATTCCGCCTAGTTGGTCACTTCGACGTATCGTCTGGAA

PCR (Q5 NEB E0555L)	JT403	CCTGTAGTCTTCTTAATTAAGACGTCAG	pGGAselect	NEB N0309AAVIAL
	JT401	GTACCAAGTCTTCGAATTCGGATC		
Blunt ligation	Enzymes used: T4 PNK (NEB M0201), DPNI (NEB R0176), T4 DNA Ligase (NEB M0202)			

#69	pGGA-JT310JT314JT311JT315			
Gibson Assembly	Primers	Sequence	Template	
PCR (Q5 NEB E0555L)	JT310	TCGAGGGATCCGAATTCGAAGACTTGGTACCGACAAGGTCTCCGTATATGAA	Lambda	NEB N3011S
	JT314	GCTAACCAATTCCTAGGCAGGTCATTG		
PCR (Q5 NEB E0555L)	JT315	CACTGTTGCCAATGACCTGCCTAG	Lambda	NEB N3011S
	JT311	TTCTGACGTCTTAATTAAGAAGACTACAGGCGACAAGGTCTCCCTGACGCTGGCATTCGCATC		
PCR (Q5 NEB E0555L)	JT403	CCTGTAGTCTTCTTAATTAAGACGTCAG	pGGAselect	NEB N0309AAVIAL
	JT401	GTACCAAGTCTTCGAATTCGGATC		
Gibson Assembly	NEB E5520 (NEB HiFi mix)			

#118	pGGA-JT298JT299 noCTCF			
KLD cloning	Primers	Sequence	Template	
PCR (Q5 NEB E0555L)	NH3	TGTGCTCAACAGACGTTTACTGTTC	#65 pGGA-JT298JT299	
	NH4	TCCTGTAATAAGCAGGGCCAG		
KLD reaction	NEB M0554S			

#65	pGGA-JT298JT299			
Blunt ligation	Primers	Sequence	Template	
PCR (Q5 NEB E0555L)	JT298	CGACAAGGTCTCGTCAGACGCGCCGGACGCTACCAGC		
	JT299	CGACAAGGTCTCGACTTCAAATCTTTCTGTATGAAGATTTGAGCACGTTGGCC	**Template GeneBlock35 (IDT):**	

GCGCCGGACGCTACCAGCTTCTTTCCCGTTGGTGGGATGCCTACCGCAAGCAGCTTGGCCTGAAAGACTTCTCTCCGAAAAGTCAGGACGCTGTGGCATTGCAGCAGATTAAGGAGCGTGGCGCTTTACCTATGATTGATCGTG
GTGATATCCGTCAGGCAATCGACCGTTGCAGCAATATCTGGGCTTCACTGCCGGGCGCTGGTTATGGTCAGTTCGAGCATAAGGCTGACAGCCTGATTGCAAAATTCAAAGAAGCGGGCGGAACGGTCAGAGAGATTGATGT
ATGAGCAGAGTCACCGCGATTATCTCCGCTCTGGTTATCTGCATCATCGTCTGCCTGTCATGGGCTGTTAATCATTACCGTGATAACGCCATTACCTACAAAGCCCAGCGCGACAAAAATGCCAGAGAACTGAAGCTGGCGAA
CGCGGCAATTACTGACATGCAGATGCGTCAGCGTGATGTTGCTGCGCTCGATGCAAAATACACGAAGGAGTTAGCTGATGCTAAAGCTGAAAATGATGCTCTGCGTGATGATGTTGCCGCTGGTCGTCGTCGGTTGCACATCA
AAGCAGTCTGTCAGTCAGTGCGTGAAGCCACCACCGCCTCCGGCGTGGATAATGCAGCCTCCCCCGACTGGCAGACACCGCTGAACGGGATTATTTCACCCTCAGAGAGAGGCTGATCACTATGCAAAAACAACTGGAAG
GAACCCAGAAGTATATTAATGAGCAGTGCAGATAGAGTTGCCCATATCGATGGGCAACTCATGCAATTATTGTGAGCAATACACACGCGCTTCCAGCGGAGTATAAATGCCTAAAGTAATAAAACCGAGCAATCCATTTACG
AATGTTTGCTGGGTTTCTGTTTTAACAACATTTTCTGCGCCGCCACAAATTTTGGCTGCATCGACAGTTTTCTTCTGCCCAATTCCAGAAACGAAGAAATGATGGGTGATGGTTTCCTTTGGTGCTACTGCTGCCGGTTTGTTTTGAA
CAGTAAACGTCTGTTGAGCACATAGCGCCCCCTGGTGGCCATCCTGTAATAAGCAGGGCCAGCGCAGTAGCGAGTAGCATTTTTTTCATGGTGTTATTCCCGATGCTTTTTGAAGTTCGCAGAATCGTATGTGTAGAAAATTAAA
CAAACCCTAAACAATGAGTTGAAATTTCATATTGTTAATATTTATTAATGTATGTCAGGTGCGATGAATCGTCATTGTATTCCCGGATTAACTATGTCCACAGCCCTGACGGGGAACTTCTCTGCGGGAGTGTCCGGGAATAATT
AAAACGATGCACACAGGGTTTAGCGCGTACACGTATTGCATTATGCCAACGCCCCGGTGCTGACACGGAAGAAACCGGACGTTATGATTTAGCGTGGAAAGATTTGTGTAGTGTTCTGAATGCTCTCAGTAAATAGTAATGA
ATTATCAAAGGTATAGTAATATCTTTTATGTTCATGGATATTTGTAACCCATCGGAAAACTCCTGCTTTAGCAAGATTTTCCCTGTATTGCTGAAATGTGATTTCTCTTGATTTCAACCTATCATAGGACGTTTCTATAAGATGCGTG
TTTCTTGAGAATTTAACATTTACAACCTTTTTAAGTCCTTTTATTAACACGGTGTTATCGTTTTCTAACACGATGTGAATATTATCTGTGGCTAGATAGTAAATATAATGTGAGACGTTGTGACGTTTTAGTTCAGAATAAAACAATT
CACAGTCTAAATCTTTTCGCACTTGATCGAATATTTCTTTAAAAAATGGCAACCTGAGCCATTGGTAAAACCTTCCATGTGATACGAGGGCGCGTAGTTTGCATTATCGTTTTTATCGTTTCAATCTGGTCTGACCTCCTTGTGTTTTGT
TGATGATTTATGTCAAATATTAGGAATGTTTTCACTTAATAGTATTGGTTGCGTAACAAAGTGCGGTCCTGCTGGCATTCTGGAGGGAAATACAACCGACAGATGTATGTAAGGCCAACGTGCTCAAATCTTCATACAGAAAGA

PCR (Q5 NEB E0555L)	JT403	CCTGTAGTCTTCTTAATTAAGACGTCAG	pGGAselect	NEB N0309AAVIAL
	JT401	GTACCAAGTCTTCGAATTCGGATC		
Blunt ligation	Enzymes used: T4 PNK (NEB M0201), DPNI (NEB R0176), T4 DNA Ligase (NEB M0202)			

#71	pGGA-JT312JT316JT313JT317			
Gibson Assembly	Primers	Sequence	Template	
PCR (Q5 NEB E0555L)	JT312	TCGAGGGATCCGAATTCGAAGACTTGGTACCGACAAGGTCTCGAAGTAAGAG	Lambda	NEB N3011S
	JT316	TTCACATAAAACATTTTGCATCAGCGAC		
PCR (Q5 NEB E0555L)	JT313	TTCTGACGTCTTAATTAAGAAGACTACAGGCGACAAGGTCTCGTGGATCCGG	Lambda	NEB N3011S
	JT317	GTGGTCAGGTTGTGGTGATTGGTC		
PCR (Q5 NEB E0555L)	JT403	CCTGTAGTCTTCTTAATTAAGACGTCAG	pGGAselect	NEB N0309AAVIAL
	JT401	GTACCAAGTCTTCGAATTCGGATC		
Gibson Assembly	NEB E5520 (NEB HiFi mix)			

#124	pGGA-CTCFdiv			
KLD cloning	Primers	Sequence	Template	
PCR (Q5 NEB E0555L)	JT444	TAGCGCCCCCTGGTGGCCAAAAGAAATCAAAGGCGCGGAC	#67	pGGA-JT294JT295
	NH2	CACGATCCCGTTTTGTGAGTTG		
KLD reaction	NEB M0554S			

#121	pBS-30-1xCTCF
Backbone	#64 pBlueScript-GoldenGate
Insert	#66 pGGA-JT292JT293
Insert	#67 pGGA-JT294JT295
Insert	#69 pGGA-JT310JT314JT311JT315
Insert	#118 pGGA-JT298JT299 noCTCF
Insert	#71 pGGA-JT312JT316JT313JT317
Golden Gate reaction	NEB E1601

#128	pBS-30-1xCTCFdiv
Backbone	#64 pBlueScript-GoldenGate
Insert	#66 pGGA-JT292JT293
Insert	#124 pGGA-CTCFdiv
Insert	#69 pGGA-JT310JT314JT311JT315
Insert	#118 pGGA-JT298JT299 noCTCF
Insert	#71 pGGA-JT312JT316JT313JT317
Golden Gate reaction	NEB E1601

Supplementary Table 2: Correction factor α and persistence length L_p for varying concentrations of Sytox Orange (SxO) and Sytox Green (SxG).

**SxO/SxG concentration [nM]**	**Correction factor α**	**Persistence length L_p [nm]**
0	1	46.1
10	1.0258	41.9
50	1.0523	36
100	1.0649	35.1
200	1.0948	37.1
500	1.3829	37.2

Figure 1a. Boxes denote areas included in the manuscript

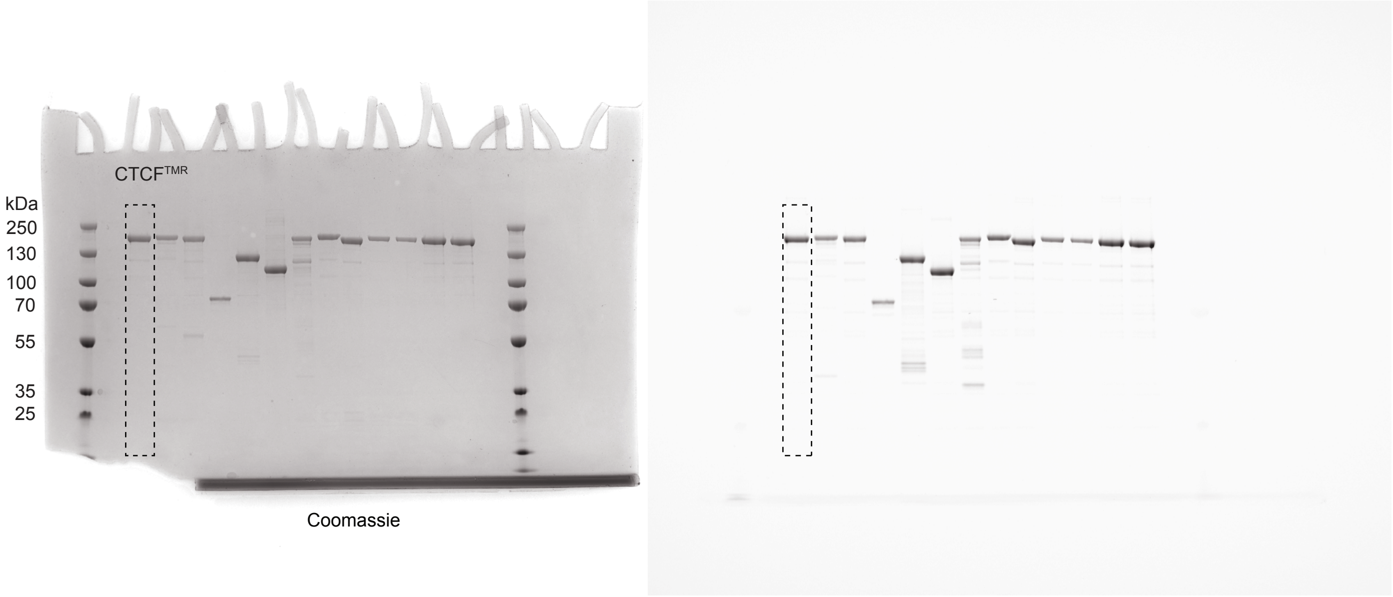

TMR

Figure 1b. Box denotes area included in the manuscript

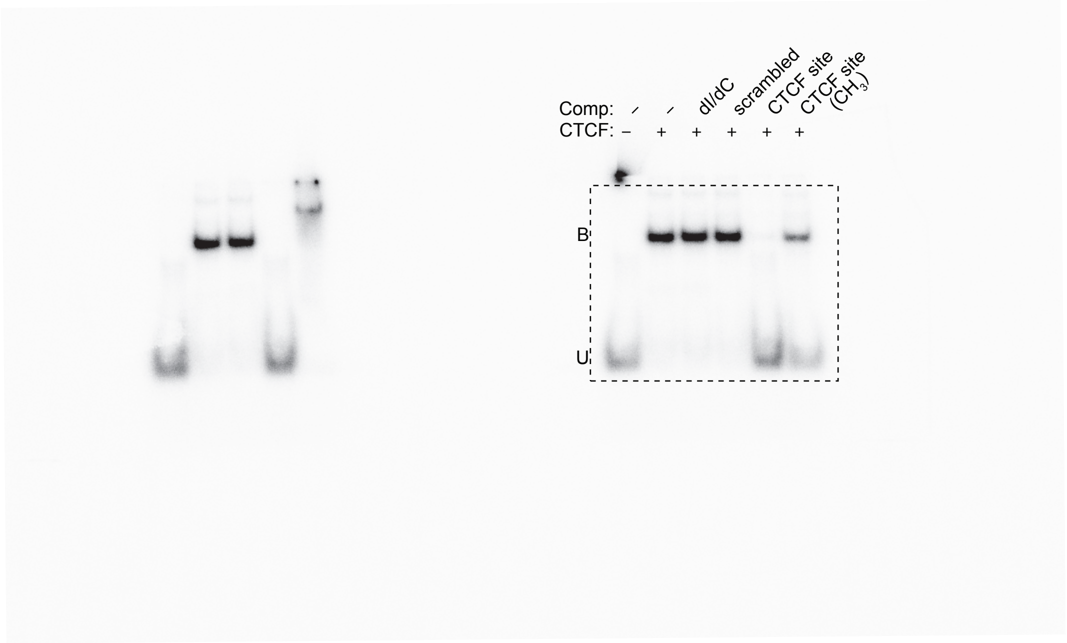

Autoradiography

Extended Data Figure 1h. Box denotes area included in the manuscript

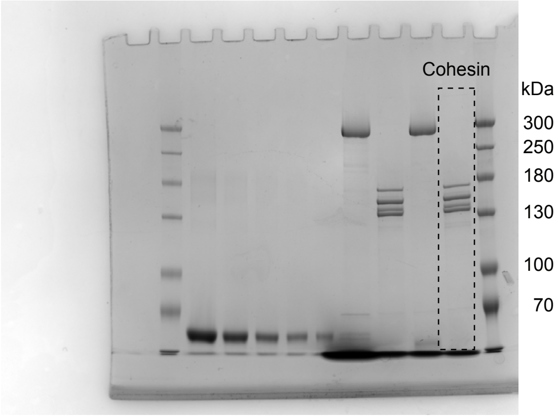

Coomassie

Extended Data Figure 1i. Box denotes area included in the manuscript

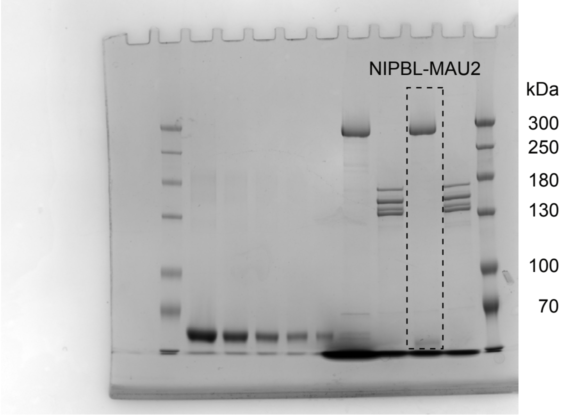

Coomassie

Extended Data Figure 3a. Boxes denote areas included in the manuscript

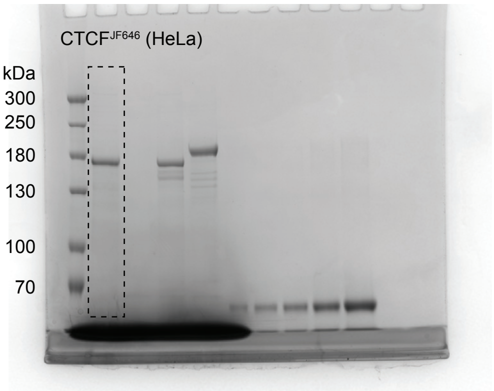

Coomassie

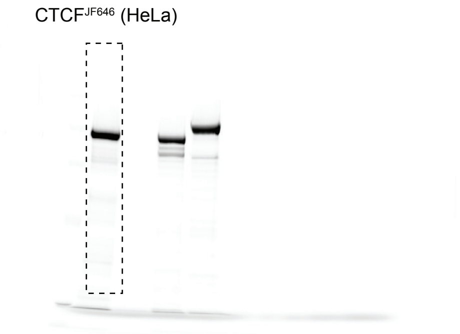

JF646

Extended Data Figure 3f. Box denotes area included in the manuscript

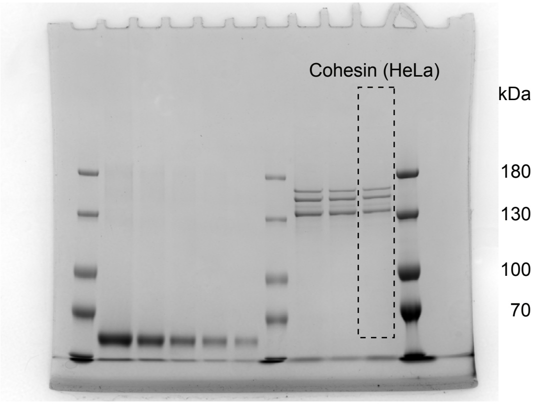

Coomassie

Supplementary Figure 1. Raw gel images.

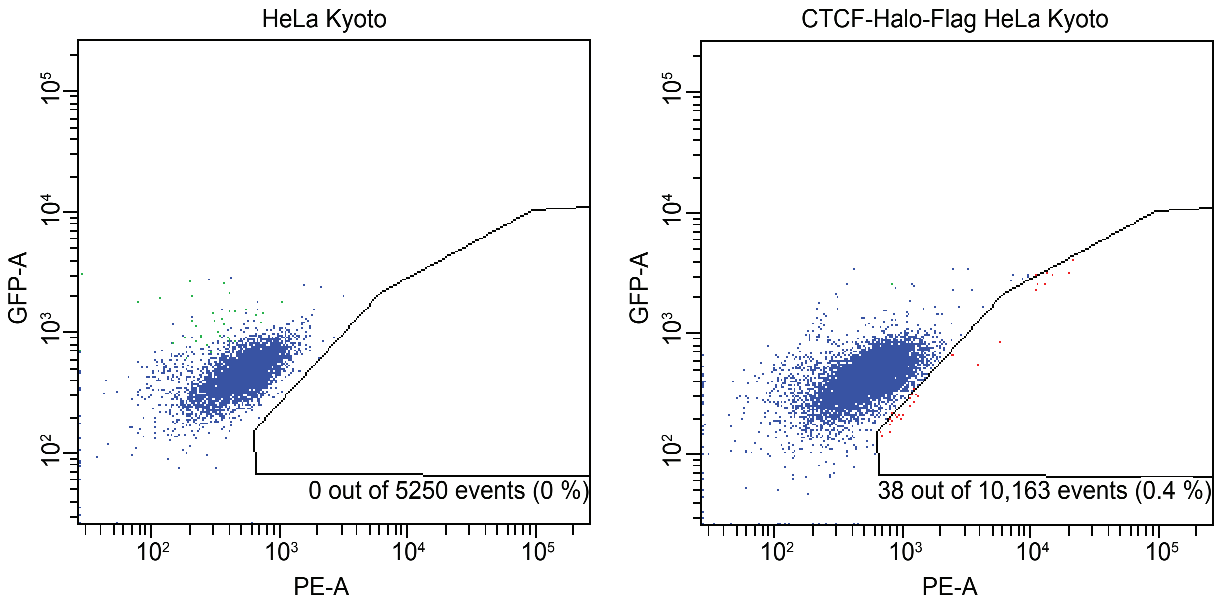

Supplementary Figure 2. FACS gating strategy for selection of cells expressing CTCF-Halo-Flag. HeLa Kyoto parental cells (left panel) or HeLa Kyoto cells transfected with donor and Cas9 nickase plasmids and incubated with Halotag TMR ligand (right panel) were sorted for PE-A (TMR) and GFP-A (autofluorescence). CTCF-Halo-Flag cells inside the gate were selected. The percentage of cells selected is denoted in the graph.
