# Supplementary material for: CTCF is a DNA-tension-dependent barrier to cohesin-mediated loop extrusion
Source: Nature. 2023 Apr 19;616(7958):822–7. doi: 10.1038/s41586-023-05961-5 (PMC10132984; doi:10.1038/s41586-023-05961-5)
Supplement: Supplementary file 2 — Reporting Summary [file 41586_2023_5961_MOESM2_ESM.pdf]

Corresponding author(s): Cees Dekker, Jan-Michael Peters

Last updated by author(s): Feb 17, 2023

Reporting Summary

Nature Portfolio wishes to improve the reproducibility of the work that we publish. This form provides structure for consistency and transparency in reporting. For further information on Nature Portfolio policies, see our Editorial Policies and the Editorial Policy Checklist.

Statistics

For all statistical analyses, confirm that the following items are present in the figure legend, table legend, main text, or Methods section.

n/a	Confirmed	
☐	☒	The exact sample size (n) for each experimental group/condition, given as a discrete number and unit of measurement
☐	☒	A statement on whether measurements were taken from distinct samples or whether the same sample was measured repeatedly
☐	☒	The statistical test(s) used AND whether they are one- or two-sided<br>*Only common tests should be described solely by name; describe more complex techniques in the Methods section.*
☒	☐	A description of all covariates tested
☒	☐	A description of any assumptions or corrections, such as tests of normality and adjustment for multiple comparisons
☐	☒	A full description of the statistical parameters including central tendency (e.g. means) or other basic estimates (e.g. regression coefficient) AND variation (e.g. standard deviation) or associated estimates of uncertainty (e.g. confidence intervals)
☐	☒	For null hypothesis testing, the test statistic (e.g. F, t, r) with confidence intervals, effect sizes, degrees of freedom and P value noted<br>*Give P values as exact values whenever suitable.*
☒	☐	For Bayesian analysis, information on the choice of priors and Markov chain Monte Carlo settings
☒	☐	For hierarchical and complex designs, identification of the appropriate level for tests and full reporting of outcomes
☒	☐	Estimates of effect sizes (e.g. Cohen's d, Pearson's r), indicating how they were calculated

Our web collection on statistics for biologists contains articles on many of the points above.

Software and code

Policy information about availability of computer code

Data collection	Zeiss Zen 3.3, Zeiss Zen 3.0 SR FP2, Micro-Manager 2.0
Data analysis	Custom python code to analyze and plot blocked fractions, DNA tension, residence times, etc. from HiLo fluorescence microscopy, as well as IGOR scripts to pre-process Magnetic Tweezer data has been deposited at https://doi.org/10.5281/zenodo.7409240. MATLAB code to analyze cohesin-mediated steps in Magnetic Tweezer data has been previously published and is accessible at https://doi.org/10.5281/<br>zenodo.4657659.<br>Python packages: numpy, scipy, trackpy, statsmodels

For manuscripts utilizing custom algorithms or software that are central to the research but not yet described in published literature, software must be made available to editors and reviewers. We strongly encourage code deposition in a community repository (e.g. GitHub). See the Nature Portfolio guidelines for submitting code & software for further information.

Data

Policy information about availability of data

All manuscripts must include a data availability statement. This statement should provide the following information, where applicable:

- Accession codes, unique identifiers, or web links for publicly available datasets
- A description of any restrictions on data availability
- For clinical datasets or third party data, please ensure that the statement adheres to our policy

All data supporting the current study are available upon reasonable request. Source data for Figures 1d, h, 2e, g, 3c-e, Extended Data Figures 1a-g, 2f, h, 3b-e, 5, 6, 7c-d, 8c-m, 9a-b, h-l, 10a-c and Supplementary Figure 3d-e are provided with the paper.

Human research participants

Policy information about studies involving human research participants and Sex and Gender in Research.

Reporting on sex and gender	*Use the terms sex (biological attribute) and gender (shaped by social and cultural circumstances) carefully in order to avoid confusing both terms. Indicate if findings apply to only one sex or gender; describe whether sex and gender were considered in study design whether sex and/or gender was determined based on self-reporting or assigned and methods used. Provide in the source data disaggregated sex and gender data where this information has been collected, and consent has been obtained for sharing of individual-level data; provide overall numbers in this Reporting Summary. Please state if this information has not been collected. Report sex- and gender-based analyses where performed, justify reasons for lack of sex- and gender-based analysis.*
Population characteristics	*Describe the covariate-relevant population characteristics of the human research participants (e.g. age, genotypic information, past and current diagnosis and treatment categories). If you filled out the behavioural & social sciences study design questions and have nothing to add here, write "See above."*
Recruitment	*Describe how participants were recruited. Outline any potential self-selection bias or other biases that may be present and how these are likely to impact results.*
Ethics oversight	*Identify the organization(s) that approved the study protocol.*

Note that full information on the approval of the study protocol must also be provided in the manuscript.

Field-specific reporting

Please select the one below that is the best fit for your research. If you are not sure, read the appropriate sections before making your selection.

☒ Life sciences ☐ Behavioural & social sciences ☐ Ecological, evolutionary & environmental sciences

For a reference copy of the document with all sections, see nature.com/documents/nr-reporting-summary-flat.pdf

Life sciences study design

All studies must disclose on these points even when the disclosure is negative.

Sample size	No statistical method was used to determine sample size. Sample size was chosen based on our previous publications on single molecule measurements. The precise number for sample size supporting respective findings are stated in the manuscript.
Data exclusions	Data in which cohesin did not encounter the roadblock (i.e. CTCF, dCas9, Au-nanoparticle) were excluded.
Replication	All experiments were performed at least twice with consistent results. We have included this information in a Statistical analysis and reproducibility section in the Methods.
Randomization	Randomisation was not relevant to this study since it did not require samples to be allocated into experimental groups.
Blinding	Each DNA template and protein required specific experimental conditions, meaning it was not possible for the investigators to be blinded during data collection.

Reporting for specific materials, systems and methods

We require information from authors about some types of materials, experimental systems and methods used in many studies. Here, indicate whether each material, system or method listed is relevant to your study. If you are not sure if a list item applies to your research, read the appropriate section before selecting a response.

Materials & experimental systems

n/a	Involved in the study	
☐	☒	Antibodies
☐	☒	Eukaryotic cell lines
☒	☐	Palaeontology and archaeology
☒	☐	Animals and other organisms
☒	☐	Clinical data
☒	☐	Dual use research of concern

Methods

n/a	Involved in the study	
☒	☐	ChIP-seq
☐	☒	Flow cytometry
☒	☐	MRI-based neuroimaging

Antibodies

Antibodies used	Digoxigenin Recombinant Rabbit Monoclonal Antibody (9H27L19), Invitrogen™
Validation	No internal validation of the antibody was done. The supplier states `No cross reactivity with other steroids, such as human estrogens e.g., estradiol or androgens e.g., testosterone.` (https://www.sigmaaldrich.com/NL/en/product/roche/11333089001)

Eukaryotic cell lines

Policy information about cell lines and Sex and Gender in Research

Cell line source(s)	Spodoptera frugiperda Sf9: Thermo Fisher Scientific.<br>HeLa Kyoto (RRID:CVCL_1922): Prof. S. Narumiya, Kyoto University, Kyoto, Japan.<br>SCC1-Halo-Flag: derived from HeLa Kyoto.<br>CTCF-Halo-Flag: derived from HeLa Kyoto.
Authentication	All HeLa Kyoto-derived cell lines were authenticated by STR fingerprinting. Sf9 cells were not authenticated.
Mycoplasma contamination	All HeLa Kyoto-derived cell lines tested negative for Mycoplasma contamination. Sf9 cells were not tested for Mycoplasma contamination.
Commonly misidentified lines<br>(See ICLAC register)	No commonly misidentified cell lines were used in this study.

Flow Cytometry

Plots

Confirm that:

☒ The axis labels state the marker and fluorochrome used (e.g. CD4-FITC).

☒ The axis scales are clearly visible. Include numbers along axes only for bottom left plot of group (a 'group' is an analysis of identical markers).

☒ All plots are contour plots with outliers or pseudocolor plots.

☒ A numerical value for number of cells or percentage (with statistics) is provided.

Methodology

Sample preparation	Cells were incubated with Halotag TMR ligand in media for 15 minutes. Cells were washed 3x in PBS and cultured for 30 minutes in media. After harvesting, cells were resuspended in PBS and immediately sorted.
Instrument	BD FACSAria III Cell Sorter
Software	BD FACSAria III Cell Sorter controlled by FACSDiva software.
Cell population abundance	1 million HeLa Kyoto / CTCF-Halo-Flag HeLa Kyoto were sorted.
Gating strategy	SSC-A vs FSC-A gating was used to filter out debris followed by SCC-W vs SCC-H gating to exclude doublets. TMR-positive cells were selected using PE-A (TMR) vs GFP-A (to exclude autofluorescence).

☒ Tick this box to confirm that a figure exemplifying the gating strategy is provided in the Supplementary Information.
